# ANN-driven analysis of Cattaneo–Christov heat transfer and entropy generation in Maxwell hybrid nanofluid flow over a vertical cone

**DOI:** 10.3389/frai.2026.1936035

**Published:** 2026-09-25

**Authors:** Bhuvana M, Durgaprasad P

**Affiliations:** Department of Mathematics, School of Advanced Sciences, Vellore Institute of Technology, Chennai, India

**Keywords:** artificial neural networks, Cattaneo-Christov heat flux, entropy generation, Maxwell fluid, non-Darcy porous medium, Sobol' sensitivity analysis

## Abstract

**Introduction:**

This research investigates Cattaneo-Christov non-Fourier heat transfer and entropy generation in Maxwell hybrid nanofluid flow over a non-Darcy porous conical surface using numerical, sensitivity, and Artificial neural networks (ANN) approaches.

**Methods:**

A coupled momentum-energy model that includes fluid relaxation, magnetic field effects, nanoparticle loading, porous resistance, and thermal relaxation is transformed using similarity variables and solved by a boundary-value numerical method. Sobol' global sensitivity analysis is employed to quantify the direct and interaction effects of the governing parameters on skin friction, Nusselt number, and entropy generation.

**Results:**

The analysis identifies the porous-medium parameter as the dominant contributor to skin friction and heat transfer, while its contribution to entropy generation remains substantial at both buoyancy conditions. The total-order indices further reveal increased parameter interactions in entropy generation at higher buoyancy. The artificial neural network, trained using the numerically generated dataset, accurately predicts skin friction, Nusselt number, and entropy generation, with test R^2^ values of 0.99479, 0.99642, and 0.99033, respectively, along with low prediction errors.

**Discussion:**

The combined numerical-Sobol' sensitivity-ANN framework enables rapid evaluation of nonlinear viscoelastic heat-transfer systems. The results underscore the utility of Maxwell hybrid nanofluids in thermally non-Fourier porous media from an engineering perspective and provide a computational framework for evaluating their thermal performance.

## 1 Introduction

Hybrid nanofluids are an enhanced form of nanofluids, which involve dispersing a combination of different nanoparticles in a base fluid to improve its thermal and physical properties. Combining various nanoparticles with pure base fluids is essential for enhancing heat transfer processes and other characteristics, resulting in widespread applications ranging from biomedical science to radiation therapy for cancer-afflicted patients, various delivery systems, diverse production industries, and microchannel power plants. Choi (1995) coined the term ‘nanofluid' to refer to a specific type of colloidal dispersion, characterized by particle sizes typically from 1 to 100 nm, composed of a liquid medium and solid nanoparticles. In biomedical applications, using blood as a carrier fluid, gold nanoparticles act as potential carriers for drug delivery, imaging agents, and the design of novel cancer treatments. Copper nanoparticles are used in many biomedical and consumer applications because of their potent antibacterial activity. Waqas et al. (2023) studied the blood flow dynamics and heat transfer in a stenosed artery with the help of gold and silver nanoparticles. Their results showed that a careful adjustment of nanoparticle characteristics could enhance the circulation and therefore, alleviate the hemodynamic changes caused by stenosis to a very great extent. Hussain et al. (2022) investigated the effect of micro- and millimeter-sized particles on blood flow through arterial stenosis. They reported that gold (Au) and silver (Ag) nanoparticles facilitate the vascular functions in stenotic arteries by lessening the shear stress, elevating the blood flow, and diminishing the formation of clots. The excellent heat conduction ability of the nanoparticles makes it feasible to reduce the risk of plaque rupture by varying the temperature, pressure, and overheating. Aljedani et al. (2025) reported that they were the first to influence gold nanoparticles' effects on blood thermal characteristics, their flow pattern in a magnetic field, and heat or mass transfer mechanisms. Zuberi et al. (2025) experimentally studied the experiments of copper-blood and alumina- blood nanofluids and found that with the volumetric fraction of nanoparticles, the temperature profile increases. This happens mainly because the nanoparticles increase heat absorption due to their increased thermal conductivities and a more extensive area to make the heat exchange. Shahzad et al. (2022) showed that adding dispersion of silver and gold nanoparticles in blood enhances its thermal conductivity and increases heat transfer rate compared to the pure base fluid during magnetohydrodynamic pulsatile flow in concentric cylinders. Zar et al. (2024) investigated MHD nanofluid flow between parallel sheets by considering Brownian motion and thermophoresis and demonstrated their significant influence on the heat and mass transfer characteristics.

Non-Newtonian fluids are essential in applied research and engineering due to their significant impact. One of the most critical subclasses of this type of fluid is a viscoelastic fluid, which exhibits both viscous and memory effects after removing applied stress. Examples include flour, dough, egg white, polymers, blood, and paints. Viscoelastic effects develop during rapid changes in strain rate linked to contractions, expansions, pulsating flows, and the onset of flow. Ahmad et al. (2024) proposed a Maxwell nanofluid model that not only facilitates heat transfer but also accurately represents the intricate flow behavior of nanoparticle-laden blood in the stenosed arteries. Gold nanoparticles, the magnetic field, and the Deborah number are the main factors that lead to the improvement of thermal efficiency and the general therapeutic effect. According to Sathyanarayana and Goud (2023), Maxwell fluid's elastic nature lowers flow velocity while encouraging greater heat storage in the boundary layer. Iranian et al. (2023) examined the behavior of Maxwell fluid over a horizontal plate under the effects of suction or injection, considering thermal radiation, and observed that increasing the Maxwell fluid parameter tends to linearize the fluid resistance behavior. Kodi et al. (2023) demonstrated that both intensified magnetic impacts and increased viscoelastic properties tend to slow down fluid movement near the cone surface. Khan et al. (2024) primarily focused on Maxwell fluid flow over a stretching sheet by considering thermal radiation. Their findings indicated that temperature and thermal boundary layer thickness rise with increased relaxation time. Temperature profiles decrease as the Maxwell fluid parameter increases, according to a study by Faridi et al. (2025) on the thermal performance of an MHD Maxwell hybrid nanofluid controlled by magnetic induction.

Recent work has continued to explore thermal transport with classical and non-Fourier heat-flux models. Parige et al. (2024) studied Fourier heat flux in a ternary hybrid nanofluid rotating over a porous disk in the presence of Joule heating. Non-Fourier formulations incorporate thermal relaxation to describe the finite speed of heat propagation, which differs from the instantaneous thermal response assumed in Fourier conduction. Crin-Barat et al. (2025) examined the relationship between the Cattaneo–Christov and Fourier heat-conduction formulations and established the relaxation of the Cattaneo–Christov system toward the classical Fourier system. More recently, Raza and Al-Zubaidi (2025) employed the Cattaneo–Christov heat-flux model to investigate non-Fourier thermal transport of nanoparticle-suspended non-Newtonian fluid over a vertical heated cone. These studies highlight the transition from classical Fourier heat conduction to relaxation-based non-Fourier formulations for describing thermal transport in complex fluid systems.

The Cattaneo-Christov model helps technology breakthroughs that improve human interactions with fundamental tasks, including food and material heating and cooling, transportation, and production. Modifying the thermal process for applications to address significant challenges over the decades in diverse fields such as electronics, power systems, thermal industries, and medicine has become essential for the viability of the Cattaneo-Christov heat flux model. Fourier's (1822) law has been employed for many years to describe heat transfer characteristics, asserting that heat transfer occurs at infinite speed. Years later, Cattaneo (1948) extended the Fourier law, claiming that heat transport occurs at a limited velocity in any medium. He introduced the time lag parameter and transformed the parabolic equation into a hyperbolic form. Subsequently, Christov (2009) derived the material invariant formulation of Cattaneo's law utilizing the Oldroyd derivative. The effective model is referred to as the Cattaneo–Christov heat flux model. Rawat et al. (2020) studied the thermally stratified nanofluid flow over a porous cone. They concluded that the nanofluid velocity diminishes as the thermal relaxation time increases, due to the material's lowered thermal conductivity. Oyelakin et al. (2017) reported that the examination of heat transfer around a vertical cone in a porous medium, employing the Cattaneo–Christov model, revealed that temperature diminishes as the thermal relaxation parameter increases, primarily due to the thermophoresis force. Zhang et al. (2021) assessed the Von Karman rotating flow of a gold-silver nanofluid in engine oil using the Cattaneo–Christov heat flux model. They noticed that increased thermal relaxation parameter values impede heat transfer among liquid particles, reducing fluid temperature, particularly in silver nanofluids. Abdeljawad et al. (2024) elucidated the influence of Cattaneo-Christov temperature and concentration fluxes, alongside Hall current and Joule heating, on the peristaltic motion of blood flow, indicating that the temperature diminishes in the Cattaneo-Christov temperature flux model relative to the conventional Fourier's law. Zaman et al. (2023) investigated the Cattaneo-Christov heat flux in stenosed and aneurysmal arteries, demonstrating that an increase in the dimensionless relaxation time parameter, which signifies the interaction duration among system parts, reduces fluid temperature.

The study of heat transfer in porous media is vital for applications including heat exchangers, thermal storage, nuclear waste management, geothermal systems, petroleum extraction, and blood circulation. Darcy's law is typically applicable to low-velocity, low-porosity flows but neglects impermeable walls, variations in porosity, and high flow rates. To resolve this, Forchheimer (1901) incorporated a nonlinear velocity term, so augmenting the characterization of non-Darcian flow. Muskat (1946) designated this word as the Forchheimer term, applicable exclusively at elevated Reynolds numbers. Zhang et al. (2025) investigated transient MHD ferrofluid natural convection in a porous cavity and demonstrated that the Darcy, Rayleigh, and Hartmann numbers significantly influence the flow and heat transfer characteristics. Saeed et al. (2021) observed that in blood-based hybrid nanofluids, an increase in the Forchheimer parameter raises fluid resistance, reducing flow over stretching and shrinking walls. Gaffar et al. (2024) established that augmenting the Forchheimer parameter, indicative of second-order inertial drag, diminishes both axial and tangential skin friction and decreases the Nusselt numbers in the flow of magnetized tangent hyperbolic fluid around a rotating cone within a non-Darcy porous medium. By increasing the non-Darcy parameter, the velocity will decrease when the pressure is kept constant in natural convection nanofluid flow across a vertical frustum cone, as per the findings by RamReddy and Venkata Rao (2017). As a result, at constant velocity, a considerable increase in the Forchheimer coefficient will elevate the pressure drop. The Forchheimer factor is the fraction which shows the contribution of fluid-solid interactions to the total pressure drop as compared to the viscous and inertial forces, based on the flow properties and heat transfer features of a viscous hybrid nanofluid in a conical gap, Al-Arabi et al.'s (2024) study. The inertia coefficient increases the mixed convection parameter and fluid velocities, but higher inertia and porosity decrease the total hybrid nanofluid speed, according to Kamal et al.'s (2025) study of heat transfer in hybrid nanofluid flow with Darcy-Forchheimer effects.

Entropy generation is used to quantify the irreversibility of fluid flow processes in thermofluid dynamic systems. An increase in entropy generation indicates greater thermodynamic irreversibility and a corresponding reduction in system efficiency. Heat transfer can become irreversible due to various thermophysical processes, including heat transfer, magnetic effects, and viscous dissipation. In numerous biological studies, analysing entropy generation supports physical and cognitive performance improvements. Evaluating entropy generation remains essential for assessing the efficiency of engineering systems. Entropy development reduces beneficial characteristics in thermal systems, leading to decreased overall performance. Enhancing the effectiveness of heat exchangers and maintaining high energy quality requires thoroughly examining entropy generation across different flow regions. Bejan (1995) performed entropy generation analysis for forced convective heat transfer and explored several parameters that reduce entropy generation in thermal systems (Bejan, 1996). Eid et al. (2024) observed that increasing the thermal relaxation parameter results in a rise in entropy and a decrease in Bejan numbers, with the impact being more significant on the Bejan numbers than on the entropy rates. Vedavathi et al. (2020) studied entropy generation in magnetohydrodynamic nanofluid flow over an inverted cone and observed that at first, Lorentz forces increase entropy near the cone surface, but as the distance from the surface increases and the magnetic influence weakens, both entropy generation and the Bejan number gradually decrease. Higher estimates of the Brinkman number boost the entropy profile in the entropy optimisation of radiative Casson fluid across a rotating cone, but the opposite impact is shown against the Bejan number, according to Deebani et al. (2020). The fluid movement in the related region generates heat when the Brinkman number is high. The Brinkman number thus enhances the entropy generation. In the study by Mehmood et al. (2022), the entropy generation increases as the cone's half-angle increases when a viscous fluid flows over a heated wavy cone in a mixed convection boundary layer flow. Li et al. (2021) analysed entropy generation in the convective flow of a viscous nanofluid over a rotating cone, accounting for heat generation and viscous dissipation, and found that increasing the diffusion parameter elevates both the entropy generation rate and Bejan number; the Brinkman number intensifies entropy production. At the same time, higher viscosity enhances the entropy rate but reduces the Bejan number. Nawaz and Arif (2022) concluded that higher Deborah numbers boost entropy generation in their numerical investigation of the MHD flow utilizing Maxwell's hybrid nanofluid.

Artificial neural networks (ANNs), a key element of soft computing, simulate how the human brain interprets and evaluates information. ANNs can compute precise answers to issues that are difficult to address analytically because of their ability to learn on their own. Researchers are actively focusing on these networks because they may be used in categorization methods. Artificial neural networks, or ANNs, depend on information moving both inside and outside the network to learn. They are both evolutionary and adaptive. Because neural network technology replicates the interconnected activity of brain cells, it may be used to learn, recognize patterns, make predictions, and overcome obstacles in a wide range of industries. Abbas et al. (2025) studied that machine learning can be a useful tool for complicated non-Newtonian circumstances in order to tackle memory-dependent transport phenomena for computer efficiency. Alqurashi and Hassan (2024) showed that an Artificial Neural Network (ANN) is a quick and dependable surrogate model that lowers computational costs and validates nonlinear heat–mass transfer simulations by exactly replicating the RKF-45 numerical solutions of radiative Williamson nanofluid flow over a porous cone with activation energy effects. Shilpa and Leela (2023) showed that the multilayer LM-based network provides high accuracy and is an effective stand-in for intricate HMT simulations by creating an ANN model trained on Crank–Nicolson FDM data to forecast heat and mass transfer rates in an inclined MHD annulus with chemical reactions and heat generation/absorption. Saidani et al. (2026) created a Levenberg-Marquardt ANN surrogate model for magneto-thermal hybrid nanofluid flow over a vertical plate and demonstrated its capacity to accurately replicate the numerical velocity and temperature solutions. Abbasi et al. (2026b) used a recurrent neural network for intelligent prediction of Darcy-Forchheimer hybrid nanofluid transport with velocity slip, nonlinear thermal radiation, porous-medium effects, and thermal stratification. Abbasi et al. (2026a) developed an ANN-LM model for the Cattaneo–Christov Carreau–Yasuda tri-hybrid nanofluid flow and showed that the model accurately predicts the velocity, temperature, and concentration profiles. More recently, Abbasi et al. (2025) applied an intelligent back-propagated neural network with the Levenberg–Marquardt algorithm to three-dimensional Carreau fluid flow under Cattaneo–Christov double diffusion and variable thermal conductivity, obtaining close agreement between the machine-learning predictions and the bvp4c reference solutions. Jalili et al. (2025) developed a deep ANN framework for predicting the thermal and flow fields of magnetized Boger-micropolar tri-hybrid nanofluids and obtained strong agreement between the ANN predictions and numerical solutions.

### 1.1 Motivation and novelty

- The thermal behavior of Maxwell viscoelastic fluids, especially blood-based hybrid nanofluids, remains insufficiently understood when exposed to magnetic fields, thermal radiation, and porous-medium resistance in conical geometries. Existing research treats Non-Fourier heat transport, entropy generation, hybrid nanoparticles, and MHD porous flow mostly in isolation, leaving a gap in understanding their combined effects on thermodynamic performance.

To address this gap, the present work:

(i) formulates a unified Maxwell–MHD–non-Darcy–radiative model with Cattaneo–Christov heat flux for a blood-based Au–Cu hybrid nanofluid flowing over a vertical cone,

(ii) provides a detailed evaluation of entropy generation and Bejan number as indicators of irreversibility,

(iii) applies a Sobol' global sensitivity analysis to identify dominant and interacting parameters governing friction, heat transfer, and entropy production, and

(iv) develops an ANN surrogate model to validate numerical results and enable fast prediction of key engineering quantities.

To clearly distinguish the present contribution from closely related Maxwell and Cattaneo–Christov studies in conical geometries, a comparative assessment is provided in Table 1.

**Table 1:** Comparative assessment of closely related studies in conical geometries.

References	Maxwell fluid	Cattaneo–Christov heat flux	MHD	Porous medium	Hybrid nanofluid	Entropy generation	ANN	Methodology
Rawat et al. (2020)	×	✓	✓	✓	×	×	×	Runge–Kutta–Fehlberg with shooting method
Oyelakin et al. (2017)	×	✓	×	✓	×	×	×	Spectral quasi-linearization method
Prabhavathi et al. (2018)	✓	×	✓	×	×	×	×	Finite element method
Kodi et al. (2023)	✓	×	✓	✓	×	×	×	Shooting method with fourth-order Runge–Kutta
**Present study**	✓	✓	✓	✓	✓	✓	✓	**MATLAB bvp4c + Sobol' analysis + ANN**

✓indicates that the corresponding physical effect or modeling feature is considered in the respective study, whereas × indicates that it is not considered. Boldface is used to highlight the present study.

This integrated numerical–sensitivity–machine-learning framework provides a systematic approach for analysing viscoelastic hybrid nanofluid transport in porous conical systems and offers useful insights into the coupled effects governing heat transfer and thermodynamic irreversibility.

## 2 Problem formulation

The current problem is about the steady flow of a Maxwell fluid around a cone using the Cattaneo–Christov model, which considers the effects of nonlinear heat conduction, porous media, and a magnetic field. The Maxwell fluid model is able to envisage delayed strain responses under complex flow scenarios by introducing a fluid relaxation time which very effectively represents the viscoelastic nature of non-Newtonian fluids. The Cattaneo–Christov heat-flux model provides a non-Fourier formulation for describing thermal transport with finite-speed heat propagation. It ensures a physically correct representation of thermal transport by removing the non-physical infinite speed of heat propagation associated with Fourier's law. Figure 1 shows the two-dimensional, steady, boundary layer heat transfer flow of a hybrid nanofluid around a vertical cone, with blood as the base fluid and gold (Au) and copper (Cu) as the dispersed nanoparticles. The coordinate system is chosen such that the x-axis is aligned with the direction of flow along the cone surface. *T*_*w*_ denotes the wall temperature and the total nanoparticle volume fraction is given by ϕ = ϕ_1_ + ϕ_2_ both are defined at the cone surface (*y* = 0). The ambient fluid temperature far from the surface is represented by *T*_∞_. A uniform magnetic field of intensity *B*_0_ is applied normal to the fluid flow direction.

**Figure 1:**
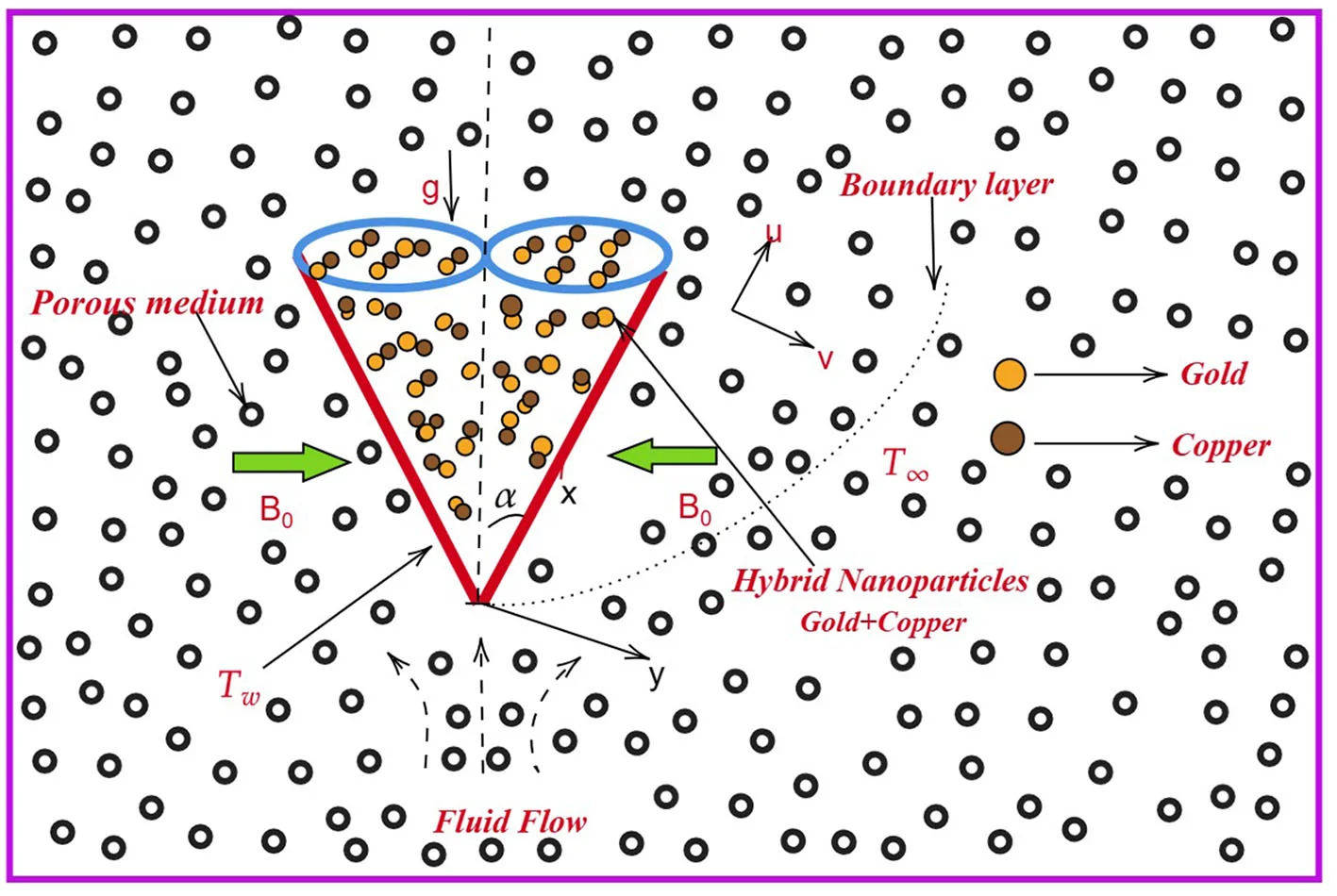
Physical configuration of the problem.

The computational procedure adopted for the numerical solution is illustrated in Figure 2. As described in Prabhavathi et al. (2018), the governing momentum and energy equations are developed by accounting for all important physical factors through a schematic description of the current flow configuration.


$$\begin{array}{l} \frac{\partial \left(ru\right)}{\partial x}+\frac{\partial \left(rv\right)}{\partial y}=0 \end{array}$$ (1)

$$\begin{array}{l} \begin{array}{c} u\frac{\partial u}{\partial x}+v\frac{\partial u}{\partial y}=\frac{\mu_{hnf}}{\rho_{hnf}}\frac{\partial^{2}u}{\partial y^{2}}-\frac{\sigma_{hnf}}{\rho_{hnf}}B^{2}u-\\ \lambda_{1}\left(u^{2}\frac{\partial^{2}u}{\partial x^{2}}+v^{2}\frac{\partial^{2}u}{\partial y^{2}}+2uv\frac{\partial^{2}u}{\partial x\partial y}\right)\\ -\frac{\mu_{hnf}}{\rho_{hnf}k_{0}}u-\frac{C_{b}}{\rho_{hnf}\sqrt{k}}u^{2}+g{\left(\rho_{\beta }\right)}_{hnf}\left(T-T_{\infty }\right) \end{array} \end{array}$$ (2)

$$\begin{array}{c} u\frac{\partial T}{\partial x}+v\frac{\partial T}{\partial y}=\frac{k_{hnf}}{{\left(\rho c_{\rho }\right)}_{hnf}}\frac{\partial^{2}T}{\partial y^{2}}-\frac{1}{{\left(\rho c_{\rho }\right)}_{hnf}}\frac{\partial q_{r}}{\partial y}-\lambda_{2}(\left(u\frac{\partial u}{\partial x}+v\frac{\partial u}{\partial y}\right)\frac{\partial T}{\partial x}\\ +\left(u\frac{\partial v}{\partial x}+v\frac{\partial v}{\partial y}\right)\frac{\partial T}{\partial y}+2uv\frac{\partial^{2}T}{\partial x\partial y}+u^{2}\frac{\partial^{2}T}{\partial x^{2}}+v^{2}\frac{\partial^{2}T}{\partial y^{2}}) \end{array}$$ (3)

**Figure 2:**
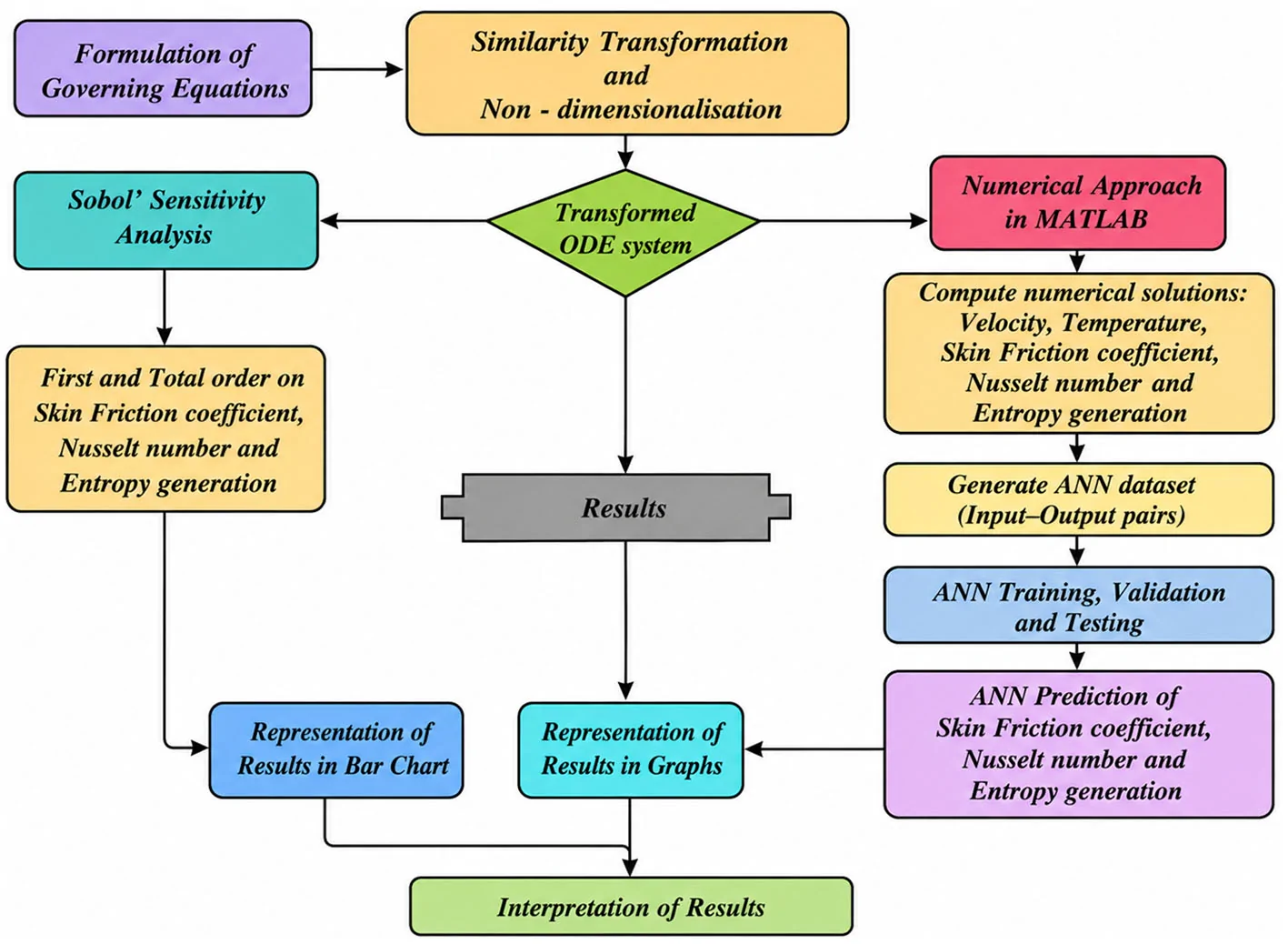
Workflow of the numerical, Sobol' sensitivity, and ANN analyses.

The boundary conditions are given in Equations 4 and 5:


$$\begin{array}{l} u=L\frac{\partial u}{\partial y}\;v=0\;T=T_{w}+K_{1}\frac{\partial T}{\partial y}\text{ at }y=0 \end{array}$$ (4)

$$\begin{array}{l} u\to 0\;T\to T_{\infty }\text{ as }y\to \infty \end{array}$$ (5)

Using the Rosseland approximation, the radiative heat flux is defined as


$$\begin{array}{l} q_{r}=-\frac{4\sigma^{*}}{3k^{*}}\frac{\partial T^{4}}{\partial y} \end{array}$$ (6)

The temperature changes in the flow are assumed to be such that the term T^4^ can be written as a linear function of temperature. Expanding *T*^4^ in a Taylor series about the free-stream temperature gives:


$$T^{4}={T_{\infty }}^{4}+4{T_{\infty }}^{3}\left(T-T_{\infty }\right)+6{T_{\infty }}^{2}{\left(T-T_{\infty }\right)}^{2}+........$$

When the higher-order terms beyond the first degree (*T* − *T*_∞_) are ignored, we obtain


$$\begin{array}{l} T^{4}≅4{T_{\infty }}^{3}T-3{T_{\infty }}^{4} \end{array}$$ (7)

Thus, substituting Equation 7 into Equation 6, we obtain the radiative heat-flux expression given in Equation 8.


$$\begin{array}{l} q_{r}=-\frac{16{T_{\infty }}^{3}\sigma^{*}}{3k^{*}}\frac{\partial T}{\partial y} \end{array}$$ (8)

The thermophysical properties are defined in Table 2 (Algehyne et al., 2025). The dynamic viscosity μ_*hnf*_, density ρ_*hnf*_, thermal diffusivity χ_*hnf*_, heat capacitance (ρ_*c*_ρ_)*hnf*_, thermal conductivity *k*_*hnf*_, and electric conductivity σ_*hnf*_ are defined in Table 3 (Mandal and Pal, 2024). The similarity transformations are as follows:


$$\begin{array}{l} \begin{array}{c} u=axf^{\prime }\left(\eta \right)\\ v=-\sqrt{a\upsilon_{f}}f\left(\eta \right)\\ \eta =\sqrt{\frac{a}{\upsilon_{f}}}y \end{array} \end{array}$$ (9)

where r can be approximated by the local radius of the cone, and is related to the x coordinate by *r* = *x*sinα. Here, *v* denotes the velocity component normal to the cone surface, whereas υ_*f*_ denotes the kinematic viscosity of the base fluid. Also, *T*_*w*_ and *T*_∞_ represent the wall and free-stream temperatures, respectively.

**Table 2:** Thermophysical features of blood Cu and Au.

Properties	Blood	Cu	Au
Specific heat capacity, $\left(C_{p}/Jkg^{-1}K^{-1}\right)$	3,594	385	129
Density, (ρ/*kgm*^−3^)	1,053	8,933	1,250
Thermal conductivity (*k*/*Wm*^−1^*K*^−1^)	0.492	401	318
Thermal expansion coefficient $\beta_{T}X10^{-5}\left(K^{-1}\right)$	0.8	1.67	1.41
Electrical conductivity (σ/*Sm*^−1^)	0.18	5.96 × 10^7^	4.1 × 10^6^

**Table 3:** Effective thermophysical-property relations for the hybrid nanofluid.

Properties	Nanofluid and HNF
Density	$\begin{array}{l} \frac{\rho_{nf}}{\rho_{f}}=\left(1-\phi_{Au}\right)+\phi_{Au}\frac{\rho_{Au}}{\rho_{blood}}\\ \frac{{\rho_{h}}_{nf}}{\rho_{f}}=\left(1-\phi_{Cu}\right)\left[\left(1-\phi_{Au}\right)+\phi_{Au}\frac{\rho_{Au}}{\rho_{blood}}\right]+\phi_{Cu}\frac{\rho_{Cu}}{\rho_{blood}} \end{array}$
Dynamic viscosity	$\begin{array}{l} \frac{\mu_{nf}}{\mu_{f}}=\frac{1}{{\left(1-\phi_{Au}\right)}^{2.5}}\\ \frac{\mu_{hnf}}{\mu_{f}}=\frac{1}{{\left(1-\phi_{Au}\right)}^{2.5}{\left(1-\phi_{Cu}\right)}^{2.5}} \end{array}$
Heat capacitance	$\begin{array}{l} \frac{{\left(\rho C_{p}\right)}_{nf}}{{\left(\rho C_{p}\right)}_{f}}=\left(1-\phi_{Au}\right)+\phi_{Au}\frac{{\left(\rho C_{p}\right)}_{Au}}{{\left(\rho C_{p}\right)}_{blood}}\\ \frac{{\left(\rho C_{p}\right)}_{hnf}}{{\left(\rho C_{p}\right)}_{f}}=\left(1-\phi_{Cu}\right)\left[\left(1-\phi_{Au}\right)+\phi_{Au}\frac{{\left(\rho C_{p}\right)}_{Au}}{{\left(\rho C_{p}\right)}_{blood}}\right]+\\ \phi_{Cu}\frac{{\left(\rho C_{p}\right)}_{Cu}}{{\left(\rho C_{p}\right)}_{blood}} \end{array}$
Electrical conductivity	$\begin{array}{l} \frac{\sigma_{nf}}{\sigma_{f}}=1-\frac{3\phi_{Au}\left[1-\frac{\sigma_{Au}}{\sigma_{blood}}\right]}{\left[2+\frac{\sigma_{Au}}{\sigma_{blood}}\right]+\phi_{Au}\left[1-\frac{\sigma_{Au}}{\sigma_{blood}}\right]}\\ \frac{\sigma_{hnf}}{\sigma_{nf}}=1-\frac{3\phi_{Cu}\left[1-\frac{\sigma_{Cu}}{\sigma_{Au}}\right]}{\left[2+\frac{\sigma_{Cu}}{\sigma_{Au}}\right]+\phi_{Cu}\left[1-\frac{\sigma_{Cu}}{\sigma_{Au}}\right]} \end{array}$
Thermal conductivity	$\begin{array}{l} \frac{K_{nf}}{K_{f}}=\frac{K_{Au}+2K_{blood}-2\phi_{Au}\left(K_{blood}-K_{Au}\right)}{K_{Au}+2K_{blood}+\phi_{Au}\left(K_{blood}-K_{Au}\right)}\\ \frac{K_{hnf}}{K_{nf}}=\frac{K_{Cu}+2K_{Au}-2\phi_{Cu}\left(K_{Au}-K_{Cu}\right)}{K_{Cu}+2K_{Au}+\phi_{Cu}\left(K_{Au}-K_{Cu}\right)}\\ \frac{K_{hnf}}{K_{f}}=\frac{K_{hnf}}{K_{nf}}X\frac{K_{nf}}{K_{f}} \end{array}$

The dimensional slip conditions are transformed using Equation 9. Since

$\frac{\partial u}{\partial y}=axf^{\prime \prime }\left(\eta \right)\;\frac{\partial \eta }{\partial y}\;=axf^{\prime \prime }\left(\eta \right)\sqrt{\frac{a}{\nu_{f}}}$

Hence, the velocity-slip condition $u=L\;\frac{\partial u}{\partial y}\;at\;y=0$, gives $f^{\prime }\left(0\right)=L\sqrt{\frac{a}{\nu_{f}}}\;f^{\prime \prime }\left(0\right),\;$ $\lambda =L\sqrt{\frac{a}{\nu_{f}}}$

Similarly, using

$\theta \left(\eta \right)=\frac{T-T_{\infty }}{T_{w}-T_{\infty }}\;$

the dimensional thermal-slip condition, $T=T_{w}+K_{1}\;\frac{\partial T}{\partial y}\;at\;y=0$ and $\frac{\partial T}{\partial y}=\left(T_{w}-T_{\infty }\right)\theta^{\prime }\left(\eta \right)\sqrt{\frac{a}{\nu_{f}}}$ gives $\theta \left(0\right)=1+K_{1}\sqrt{\frac{a}{\nu_{f}}}\theta^{\prime }\left(0\right),\;$ $\xi =K_{1}\sqrt{\frac{a}{\nu_{f}}}$

The impermeability condition gives

*v*(0) = 0 ⇒ *f*(0)= 0, while the conditions *u* → 0, *T* → *T*_∞_ *as y* → ∞ transform to

*f*′(η) → 0, θ(η) → 0 *as η* → ∞

Substituting Equation 9 into Equations 1–3, we obtain the nonlinear ordinary differential equations given in Equations 10 and 11.


$$\begin{array}{l} \begin{array}{c} f^{\prime \prime \prime }-A_{1}A_{2}\left({f^{\prime }}^{2}-ff^{\prime \prime }\right)+A_{1}A_{2}\Lambda \left(2ff^{\prime }f^{\prime \prime }-f^{2}f^{\prime \prime \prime }\right)-\\ F^{*}A_{2}f^{\prime 2}-A_{2}A_{5}Mf^{\prime }-Kf^{\prime }+A_{1}A_{2}Gr\theta =0 \end{array} \end{array}$$ (10)

$$\begin{array}{l} \left(A_{4}+R\right)\theta^{\prime \prime }+\mathrm{Pr}A_{3}f\theta^{\prime }-\mathrm{Pr}\beta A_{3}\left(ff^{\prime }\theta^{\prime }+f^{2}\theta^{\prime \prime }\right)=0 \end{array}$$ (11)

The transformed boundary conditions are given in Equation 12:


$$\begin{array}{l} f\left(0\right)=0,\;f^{\prime }\left(0\right)=\lambda f^{\prime \prime }\left(0\right)\;\theta \left(0\right)=1+\xi \theta^{\prime }\left(0\right) \end{array}$$

$$\begin{array}{l} f^{\prime }\left(\eta \right)\to 0\;\theta \left(\eta \right)\to 0\text{ as }\eta \to \infty \end{array}$$ (12)

where prime denotes differentiation with respect to η, and the significant thermophysical parameters dictating the flow dynamics are defined by


$$\begin{array}{l} M=\frac{\sigma_{f}B^{2}}{a\rho_{f}}\;\Lambda =a\lambda_{1}\;F^{*}=\frac{C_{b}x}{\rho_{f}\sqrt{k}}\;K=\frac{\upsilon_{f}}{ak_{0}}\\ Gr=\frac{g{\left(\rho_{\beta }\right)}_{hnf}\left(T_{w}-T_{\infty }\right)\cos\alpha }{a^{2}x} \end{array}$$

$$\begin{array}{l} R=\frac{16\sigma^{*}{T_{\infty }}^{3}}{3k^{*}k_{f}}\;\mathrm{Pr}=\frac{\upsilon_{f}{\left(\rho c_{\rho }\right)}_{f}}{k_{f}}\;\lambda =L\sqrt{\frac{a}{\upsilon_{f}}}\;\xi =K_{1}\sqrt{\frac{a}{\upsilon_{f}}}\;\\ \beta =\lambda_{2}a \end{array}$$

$$\begin{array}{l} A_{1}={\left(1-\phi_{1}\right)}^{2.5}{\left(1-\phi_{2}\right)}^{2.5}\\ \left(\left(1-\phi_{2}\right)\left(\left(1-\phi_{1}\right)+\phi_{1}\frac{\rho_{Ag}}{\rho_{f}}\right)+\phi_{2}\frac{\rho_{Au}}{\rho_{f}}\right) \end{array}$$

$$\begin{array}{l} A_{2}={\left(1-\phi_{1}\right)}^{2.5}{\left(1-\phi_{2}\right)}^{2.5} \end{array}$$

$$\begin{array}{l} A_{3}=\left(1-\phi_{2}\right)\left(\left(1-\phi_{1}\right)+\phi_{1}\frac{{\left(\rho c_{\rho }\right)}_{Ag}}{{\left(\rho c_{\rho }\right)}_{f}}\right)+\phi_{2}\frac{{\left(\rho c_{\rho }\right)}_{Ag}}{{\left(\rho c_{\rho }\right)}_{f}} \end{array}$$

$$\begin{array}{l} A_{4}=\frac{k_{hnf}}{k_{f}} \end{array}$$

$$\begin{array}{l} A_{5}=\frac{\sigma_{hnf}}{\sigma_{f}} \end{array}$$

Quantities of practical interest in this problem are the skin-friction coefficient *C*_*f*_*x*__, and the local Nusselt number *Nu*_*x*_. These quantities are defined in Equation 13.


$$\begin{array}{l} \;\begin{array}{c} C_{f_{x}}=\frac{\tau_{w}}{\rho_{f}U_{\infty }^{2}},\\ Nu_{x}=\frac{xq_{w}}{k\left(T_{w}-T_{\infty }\right)} \end{array}\} \end{array}$$ (13)

The non-dimensional expressions for the skin friction and Nusselt number in terms of similarity transformation are given in Equation 14.


$$\begin{array}{l} \;\begin{array}{c} R_{e_{x}}^{1/2}C_{f_{x}}=A_{1}\left(1+\Lambda \right)f^{\prime \prime }\left(0\right)\\ R_{e_{x}}^{-1/2}Nu_{x}=-\left(A_{4}+R\right)\theta^{\prime }\left(0\right) \end{array}\} \end{array}$$ (14)

where $R_{e_{x}}=\frac{U_{w}x}{\upsilon_{f}}$

## 3 Entropy generation

The entropy model for magneto-fluid with porosity effects can be described is given in Equation 15 Ramzan et al. (2019).


$$\begin{array}{l} {S^{\prime \prime \prime }}_{gen}=\underset{HeatFrictionIrreversibility}{\underset{︸}{\frac{1}{T_{\infty }}\left[k_{hnf}+\frac{16{T_{\infty }}^{3}\sigma^{*}}{3k^{*}}\right]{\left(\frac{\partial T}{\partial y}\right)}^{2}+\frac{\mu_{hnf}}{T_{\infty }}{\left(\frac{\partial u}{\partial y}\right)}^{2}}} \end{array}$$

$$\begin{array}{r} +\underset{FluidFrictionIrreversibility}{\underset{︸}{\frac{\sigma_{hnf}}{T_{\infty }}Bu^{2}+\frac{\mu_{hnf}}{T_{\infty }k_{0}}u^{2}}} \end{array}$$ (15)

Here, fluid friction is denoted by FFI and heat transfer-generated entropy by HFI. Entropy generation due to viscous dissipation is the first term in FFI; entropy generation due to magnetic field effects is the second term; and entropy generation due to the porosity effects is the third term.

The ratio of the volumetric entropy generation (${S^{\prime \prime \prime }}_{gen}$) to the characteristic entropy rate (*S*_0_) is known as the dimensionless entropy generation variable and is given in Equation 16.


$$\begin{array}{l} E_{G}=\frac{{S^{\prime \prime \prime }}_{gen}}{S_{0}} \end{array}$$ (16)

The entropy heat generation is therefore written as Equation 17


$$\begin{array}{l} E_{G}=\left(A_{4}+R\right)\alpha_{1}\theta^{\prime 2}+\frac{Br}{A_{2}}f^{\prime \prime 2}+A_{5}MBrf^{\prime 2}+\frac{Br}{A_{2}}Kf^{\prime 2} \end{array}$$ (17)

where $\alpha_{1}=\frac{T_{f}-T_{\infty }}{T_{\infty }}=\frac{\Delta T}{T_{\infty }}$ and $Br=\frac{\mu_{f}{\left(ax\right)}^{2}}{k_{f}\left(T_{f}-T_{\infty }\right)}$

One important metric to assess the irreversibility distribution is the Bejan number (*N*_*G*_). It is defined as the ratio of entropy generation caused by heat transfer to total entropy generation.

Mathematically, the Bejan number is given in Equation 18.


$$\begin{array}{l} N_{G}\;=\;\frac{Thermal\;Irreversibilty}{Total\;Irreversibility} \end{array}$$

$$\begin{array}{l} N_{G}=\frac{\left(A_{4}+R\right)\alpha_{1}\theta^{\prime 2}}{A_{4}\left(1+R\right)\alpha_{1}\theta^{\prime 2}+\frac{Br}{A_{2}}f^{\prime \prime 2}+A_{5}MBrf^{\prime 2}+\frac{Br}{A_{2}}Kf^{\prime 2}} \end{array}$$ (18)

## 4 Numerical solution

### 4.1 Solution setup

Using the bvp4c approach in MATLAB, we reduced the higher-order ODEs (10–11) to first-order ODEs, which we subsequently solved numerically. The following transformations are given in Equations 19 and 20.


$$\begin{array}{l} y=\left[f,f^{\prime },f^{\prime \prime },\theta ,\theta^{\prime }\right] \end{array}$$ (19)

$$\begin{array}{l} f=y_{1},f^{\prime }=y_{2},f^{\prime \prime }=y_{3},\theta =y_{4},\theta^{\prime }=y_{5} \end{array}$$ (20)

The converted first-order system is given in Equations 21 and 22:


$$\begin{array}{l} y_{3}\prime =\frac{1}{1+A_{1}A_{2}\Lambda {y_{1}}^{2}}\left(\begin{array}{l} \left(A_{1}A_{2}\right)\left({y_{2}}^{2}-y_{1}y_{3}\right)-2A_{1}A_{2}\Lambda y_{2}y_{3}+\\ F^{*}A_{2}{y_{2}}^{2}+A_{2}A_{5}My_{2}-A_{1}A_{2}Gry_{4} \end{array}\right) \end{array}$$ (21)

$$\begin{array}{l} {y_{5}}^{\prime }=\frac{1}{A_{4}+R-\mathrm{Pr}\beta A_{3}{y_{1}}^{2}}\left(-PrA_{3}y_{1}y_{5}+Pr\beta A_{3}y_{1}y_{3}y_{5}\right) \end{array}$$ (22)

The corresponding boundary conditions are:


$$\begin{array}{l} y_{1}\left(0\right)=0,y_{2}\left(0\right)=\lambda y_{3}\left(0\right),y_{4}\left(0\right)=1+\varepsilon y_{5}\left(0\right)\\ y_{2}\left(\infty \right)=0,y_{4}\left(\infty \right)=0 \end{array}$$

### 4.2 Algorithm

- The first step is to change the original higher-order nonlinear ordinary differential equations that describe heat and fluid movement into a set of first-order ODEs.
- The similarity variable η will range from 0 to a suitable upper limit, usually between 6 and 8, which represents an infinite boundary layer domain.
- We provide the solver with an initial guess for the temperature and velocity profiles to help it obtain the numerical solution.
- The solution is then obtained using MATLAB's bvp4c solver for handling nonlinear boundary-value problems. The solver instructions include:
  – A function that the system of first-order ODEs
  – A function that determines the boundary conditions at η = 0 and η → ∞, and
  – A structure created by bvpinit that specifies the initial profile and mesh points.
- To facilitate convergence and enhance the solution's stability, the Jacobian matrix of the system can be supplied as an optional input. Numerical stability is monitored, and adjustments to initial guesses or mesh refinement are made if required.
- Continuation techniques are implemented by updating parameter values, using previously obtained solutions as initial guesses. This ensures computational efficiency and robust solver performance.
- To obtain important physical quantities such as wall shear stress (skin-friction coefficient) and wall heat flux (Nusselt number), the derivatives of velocity and temperature are evaluated at η = 0.
- The mesh is refined until further changes do not significantly affect the results, thereby ensuring grid independence.
- Graphs and tables effectively present the results, as they clearly illustrate how different factors influence flow and heat transfer.

### 4.3 Code validation

To verify the accuracy of the numerical implementation, the MATLAB bvp4c procedure was benchmarked against the results reported by Khan et al. (2014) for water-based single-wall carbon nanotube (SWCNT) and multi-wall carbon nanotube (MWCNT) nanofluids. The benchmark governing equations, boundary conditions, and thermophysical properties were adopted with Pr = 6.2, zero velocity slip, and nanoparticle volume fractions ϕ = 0.01, 0.10, and 0.20. The reduced skin-friction coefficient, ${R_{e_{x}}}^{1/2}C_{f_{x}}$, and the reduced Nusselt number, ${R_{e_{x}}}^{-1/2}Nu_{x}$, computed using bvp4c are compared with the published values in Table 4.

**Table 4:** Comparison of the present bvp4c results with Khan et al. (2014).

ϕ	Nanofluid	${R_{e_{x}}}^{1/2}C_{f_{x}}$		${R_{e_{x}}}^{-1/2}Nu_{x}$	
		Khan et al.	Present bvp4c	Khan et al.	Present bvp4c
0.01	SWCNT-water	0.33894	0.33899	1.10553	1.10558
0.10	SWCNT-water	0.40811	0.40826	4.80627	4.80684
0.20	SWCNT-water	0.50452	0.50517	12.30317	12.30845
0.01	MWCNT-water	0.33727	0.33732	1.07905	1.07910
0.10	MWCNT-water	0.39008	0.39036	4.27718	4.27821
0.20	MWCNT-water	0.46466	0.46638	10.56783	10.58089

The computed values agree closely with the benchmark results for both SWCNT–water and MWCNT–water nanofluids. The largest percentage difference is 0.3706%, occurring for the reduced skin-friction coefficient of MWCNT–water at ϕ = 0.20, while the differences for the other cases are smaller. The close agreement between the two sets of results supports the numerical accuracy of the bvp4c implementation used in the present study.

### 4.4 Mesh sensitivity and solution convergence analysis

A numerical sensitivity and convergence assessment was performed using a representative parameter combination within the ranges considered in the numerical analysis. The physical parameters were fixed as


$$Gr\;=\;0.5,\;M\;=\;1,\;K\;=\;2,\;F^{*}=\;2,\;R\;=\;1,\;\lambda \;=\;0.4,\;\xi \;=\;0.4,$$

$$\Lambda \;=\;3.1,\;\beta \;=\;0.1,\;Pr\;=\;21,\;\varphi_{Au}\;=\;\varphi_{Cu}\;=\;0.02.$$

First, the sensitivity to the initial mesh was examined using 100, 200, 400, and 800 mesh points at a fixed computational domain and solver tolerance. Since bvp4c employs adaptive mesh refinement, the test evaluates the influence of the initial mesh selection. The computed wall quantities remained unchanged to the reported precision for all initial mesh sizes as shown in Table 5.

**Table 5:** Initial-mesh sensitivity analysis.

Initial mesh points	Final adaptive nodes	f′(0)	f″(0)	–θ′(0)
100	353	0.04507706	0.11269265	0.48760318
200	390	0.04507706	0.11269265	0.48760318
400	411	0.04507706	0.11269265	0.48760318
800	647	0.04507706	0.11269265	0.48760318

The relative changes between successive initial meshes were of the order of


$${10^{-}}^{9}\;\%\;-\;{10^{-}}^{10}\%,$$

showing that the numerical solution is insensitive to the initial mesh selection. The wall-slip condition was also satisfied for every mesh.


$$f^{\prime }\left(0\right)\;=\;\lambda \;f^{\prime \prime }\left(0\right).$$

In addition, solver-tolerance and computational-domain convergence were examined while all physical parameters were kept unchanged. The corresponding results are listed in Table 6.

**Table 6:** Solver-tolerance and computational-domain convergence.

Convergence test	Numerical setting	f″(0)	–θ′(0)
Solver tolerance	10^−4^ / 10^−6^	0.11269265	0.48760318
	10^−5^ / 10^−7^	0.11269265	0.48760318
	10^−6^ / 10^−8^	0.11269265	0.48760318
	10^−7^ / 10^−9^	0.11269265	0.48760318
Computational domain	*η_∞_*= 6	0.11266771	0.48773935
	*η_∞_*= 8	0.11269265	0.48760318
	*η_∞_*= 10	0.11269426	0.48759444
	*η_∞_*= 12	0.11269436	0.48759387

The wall quantities remained unchanged to the reported precision under successive solver-tolerance refinement. Extending the computational domain from η_∞_ = 10 to η_∞_ = 12 changed f″ (0) and –θ′ (0) by only 0.000092% and 0.000116%, respectively. The mesh-sensitivity, tolerance-refinement, and domain-extension results therefore demonstrate the numerical stability and convergence of the boundary-value solution.

## 5 Results and discussions

In the dimensionless model, *Gr* = 0.5 and *Gr* = 1.5 are selected to represent two levels of buoyancy, where the lower value corresponds to a relatively smaller buoyancy contribution and the higher value corresponds to a greater influence of buoyancy on the flow. Thus, the effects of the governing parameters are compared under different buoyancy conditions while the remaining baseline parameters are kept unchanged.

Figure 3 depicts the velocity profile of the hybrid nanofluid for different values of the magnetic parameter M. It can be observed that the velocity decreases with increasing M for both *Gr* = 0.5 and *Gr* = 1.5. Physically, increasing M strengthens the Lorentz force, which acts as a resistive force against fluid motion and reduces momentum within the boundary layer. At a fixed M, the velocity is higher for *Gr* = 1.5 because the larger buoyancy force assists the upward motion of the heated fluid along the vertical cone. Therefore, the velocity distribution is governed by the competition between magnetic resistance and buoyancy-driven flow. The increase in the magnetic parameter M increases the temperature profile, as shown in Figure 4. The Lorentz force generated by the magnetic field impedes fluid motion and reduces convective heat transfer, which causes more thermal energy to remain within the boundary layer, thus increasing its temperature. Figures 5, 6 show the variations in velocity and temperature for different values of the porosity parameter K. An increase in the porous resistance parameter increases the opposition to fluid motion, which leads to a reduction in velocity. The temperature profiles increase with increasing values of the porosity parameter, whereas the velocity profiles exhibit the opposite behavior. The porosity parameter increases the thermal boundary-layer thickness by generating a resistance force against the flow field (Elbashbeshy and Asker, 2022). The resulting reduction in fluid motion weakens convective heat transfer and, consequently, increases the temperature and thermal boundary-layer thickness.

**Figure 3:**
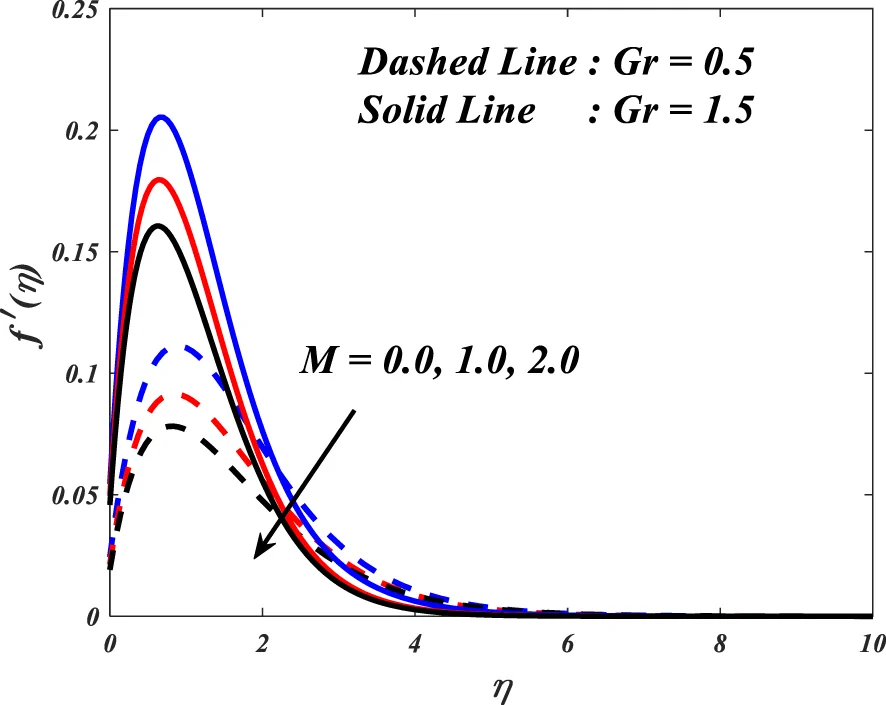
Influence of magnetic parameter M on velocity distribution.

**Figure 4:**
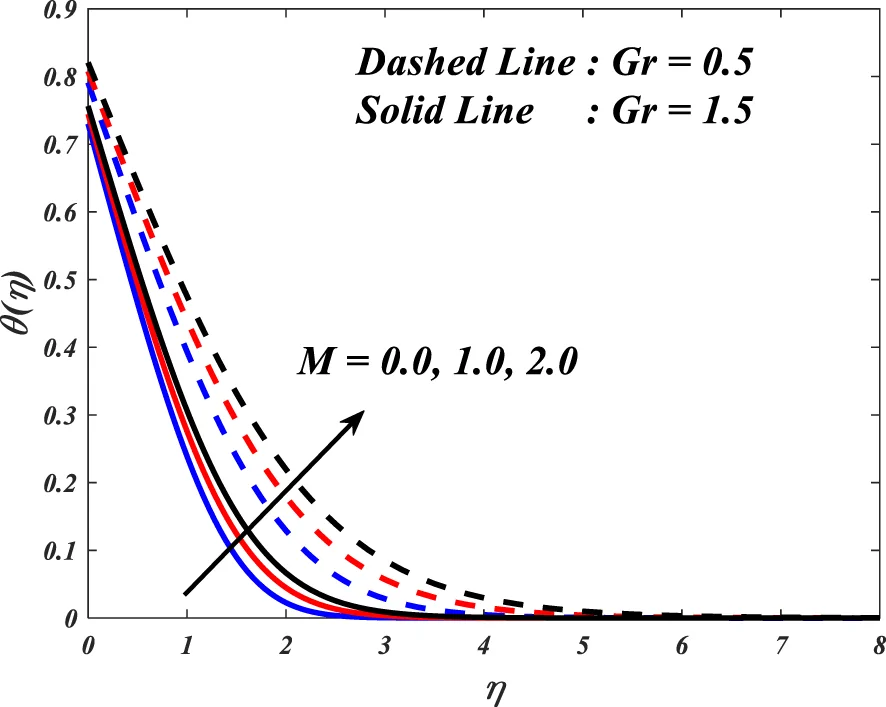
Influence of the magnetic parameter M on the temperature distribution.

**Figure 5:**
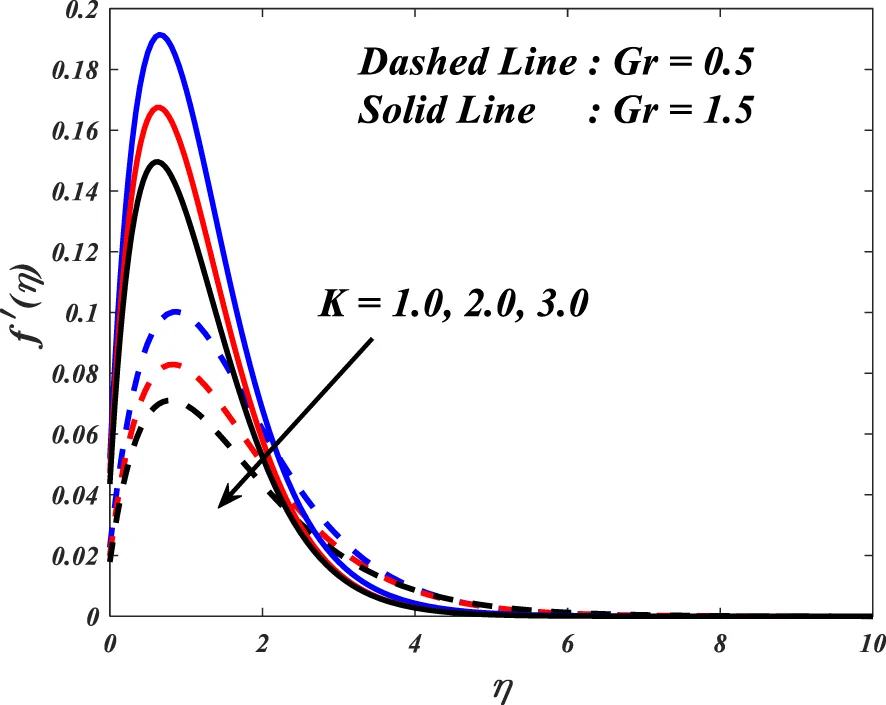
Influence of the porosity parameter K on the velocity distribution.

**Figure 6:**
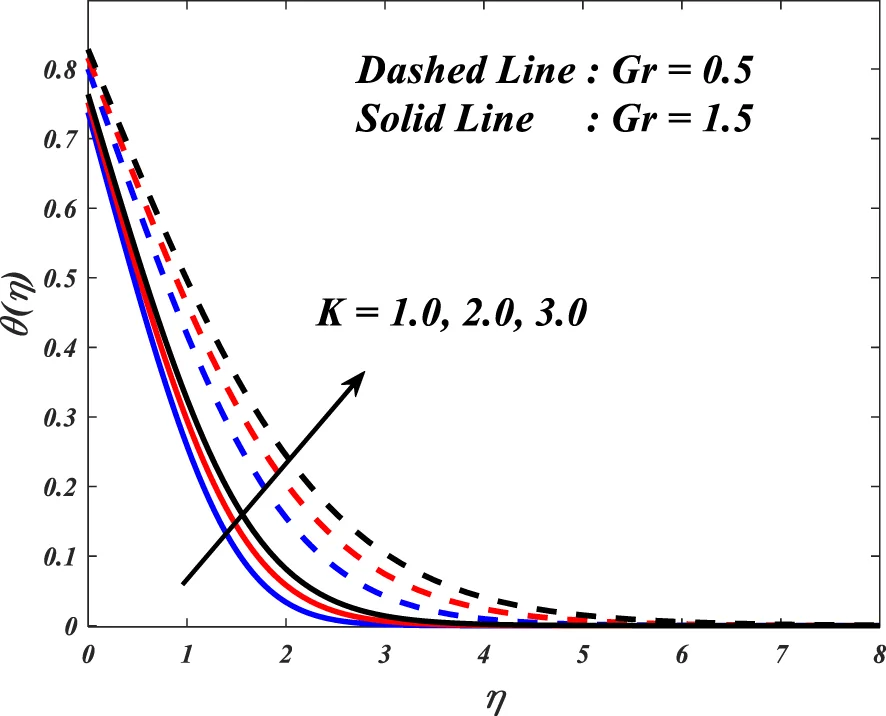
Influence of porosity parameter K on temperature distribution.

The velocity profile along a cone decelerates due to the local inertia coefficient, which is commonly expressed as F^*^ in Darcy-Forchheimer flow. Fluid velocity decreases close to the cone surface as the inertia coefficient rises. It slows down because the fluid motion is resisted by inertial forces, which are directly related to the inertia coefficient in both cases of the Grashof number *Gr* = 0.5 and *Gr* = 1.5 as shown in Figure 7. Physically, a larger F^*^ strengthens the nonlinear inertial resistance of the porous medium, which extracts momentum from the flow and suppresses the velocity within the boundary layer. An improved temperature distribution results from increasing the radiation parameter, as shown in Figure 8. A higher radiation parameter increases the radiative contribution to thermal energy transport, which raises the fluid temperature within the boundary layer. As a result, the thermal boundary layer thickens as R increases. Figure 9 shows that the fluid velocity inside the boundary layer rises as the velocity slip parameter increases. This occurs because the fluid travels more rapidly due to decreased friction from slip at the cone surface. As the velocity slip parameter increases. Figure 10 shows that the thermal boundary layer thickness decreases as the thermal slip parameter increases. This behavior is explained by the fact that when thermal slip increases, the thermal contact between the cone surface and the boundary layer fluid decreases. A thinner thermal boundary layer is created as a result of less heat being transmitted from the cone to the fluid, thus lowering the temperature inside the boundary layer. The velocity profile across a cone is significantly influenced by the Deborah number, which particularly affects the thickness of the momentum boundary layer. Figure 11 illustrates how the velocity components decrease as the Deborah number Λ increases. This is expected as increasing Λ lengthens the fluid's relaxation time, thereby strengthening the viscoelastic response and increasing the resistance to deformation, which suppresses fluid motion within the momentum boundary layer. The lower Deborah number considered in the present analysis, Λ = 0.1, represents a weak viscoelastic relaxation regime and is considered in accordance with the Maxwell-type relaxation behavior reported for blood (Herrera-Valencia et al., 2024). The higher values, Λ = 3.1 and 6.1 are considered as parametric extensions to examine the flow response under stronger viscoelastic relaxation effects rather than as direct physiological values.

**Figure 7:**
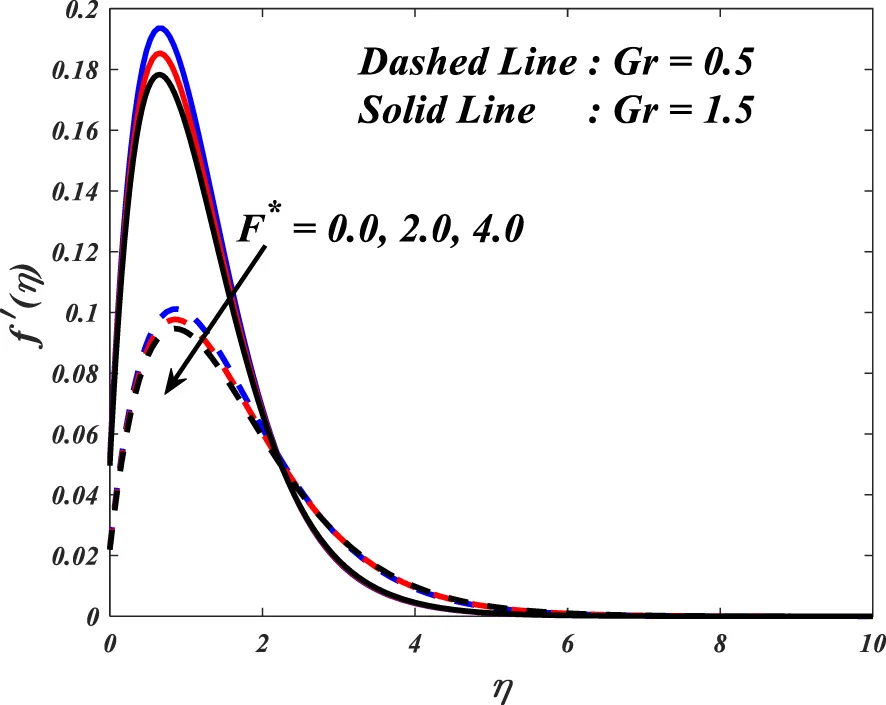
Influence of the inertia coefficient F* on the velocity distribution.

**Figure 8:**
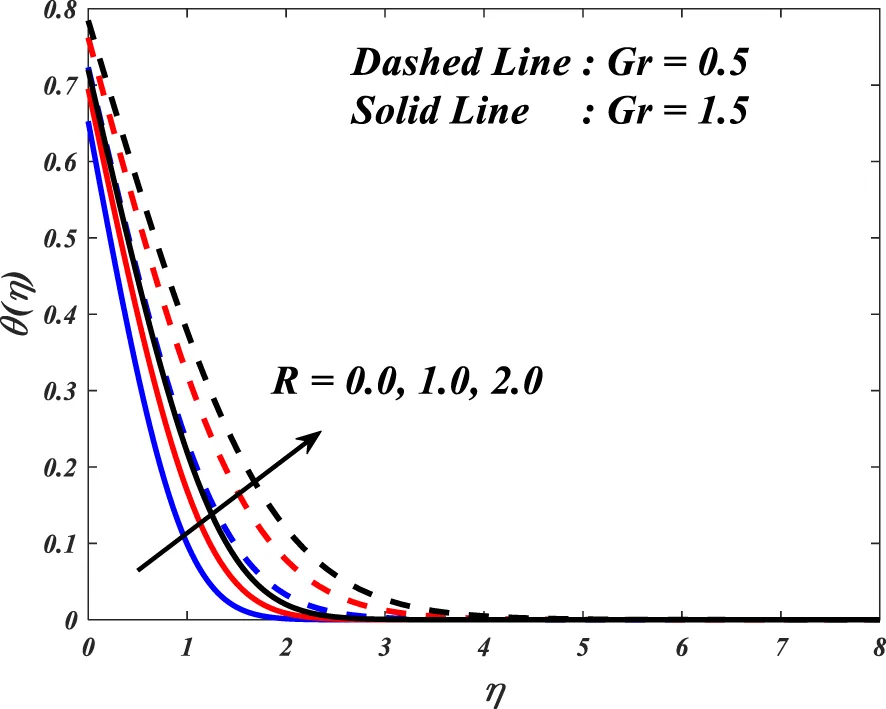
Influence of the radiation parameter on the temperature distribution.

**Figure 9:**
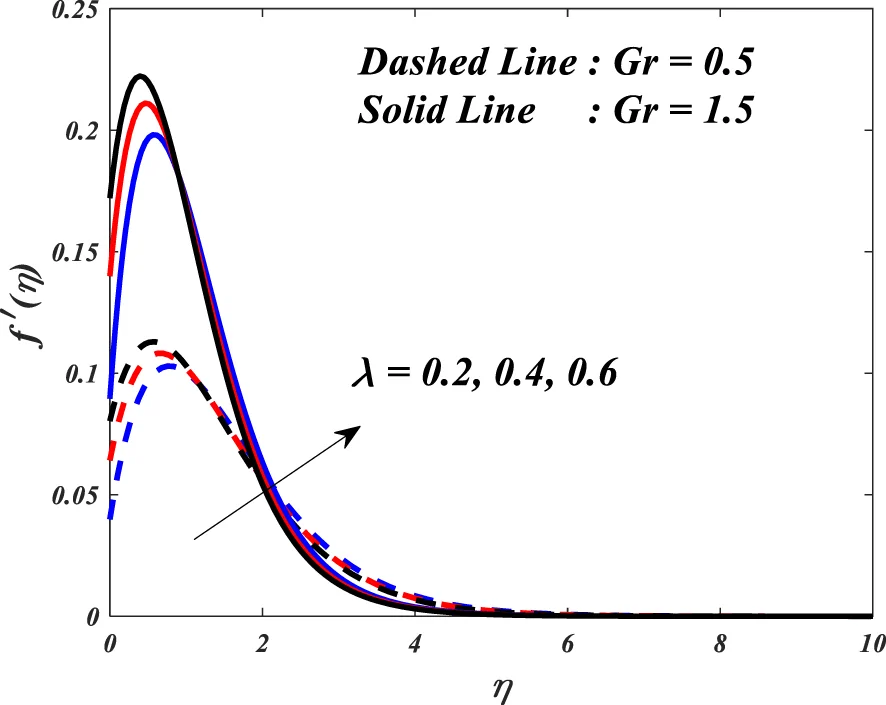
Influence of the velocity slip parameterλ on the velocity distribution.

**Figure 10:**
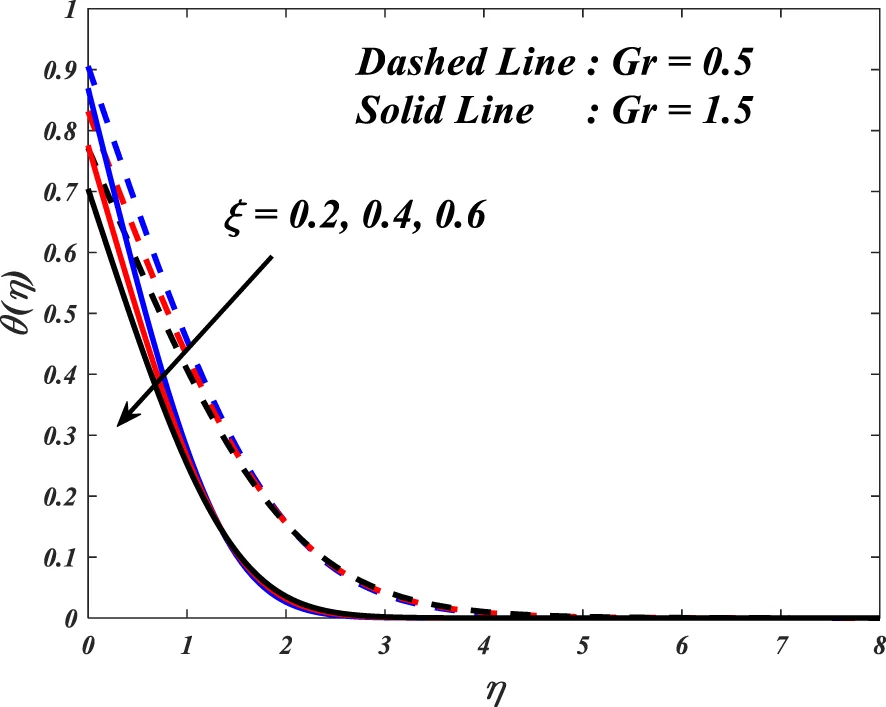
Influence of the temperature slip parameter ξ on the temperature distribution.

**Figure 11:**
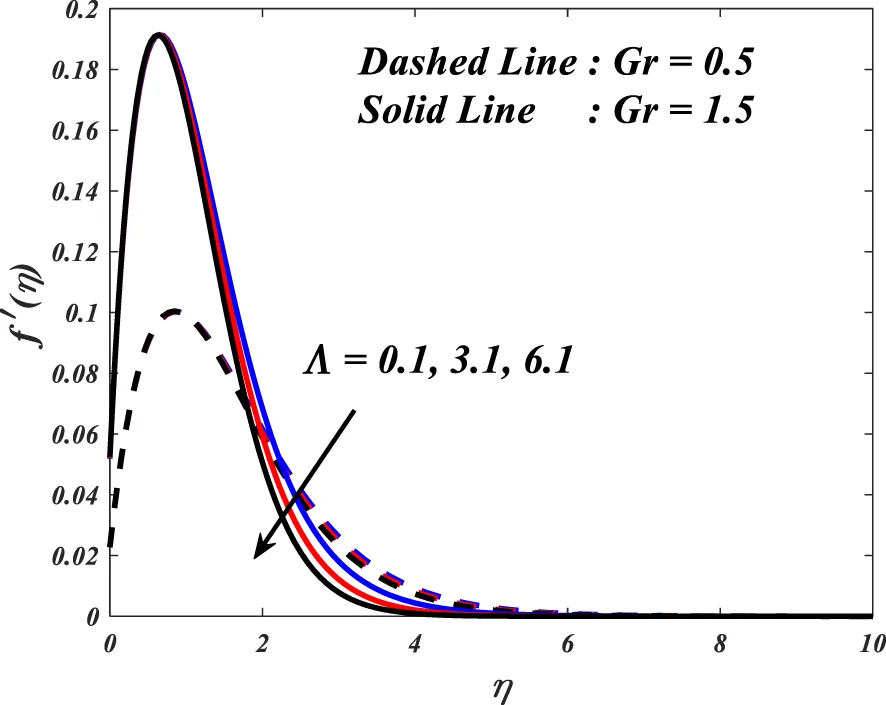
Influence of the Deborah number Λ on velocity distribution.

The temperature changes for larger values of the thermal relaxation parameter β are shown in Figure 12. It is noted that as β rises, θ (η) decreases. In physical terms, a larger β indicates that it takes longer for the fluid particles to transfer heat to their surroundings. A larger thermal relaxation time delays the response of the heat flux to the temperature gradient, thereby reducing thermal energy propagation through the boundary layer and decreasing the temperature profile.

**Figure 12:**
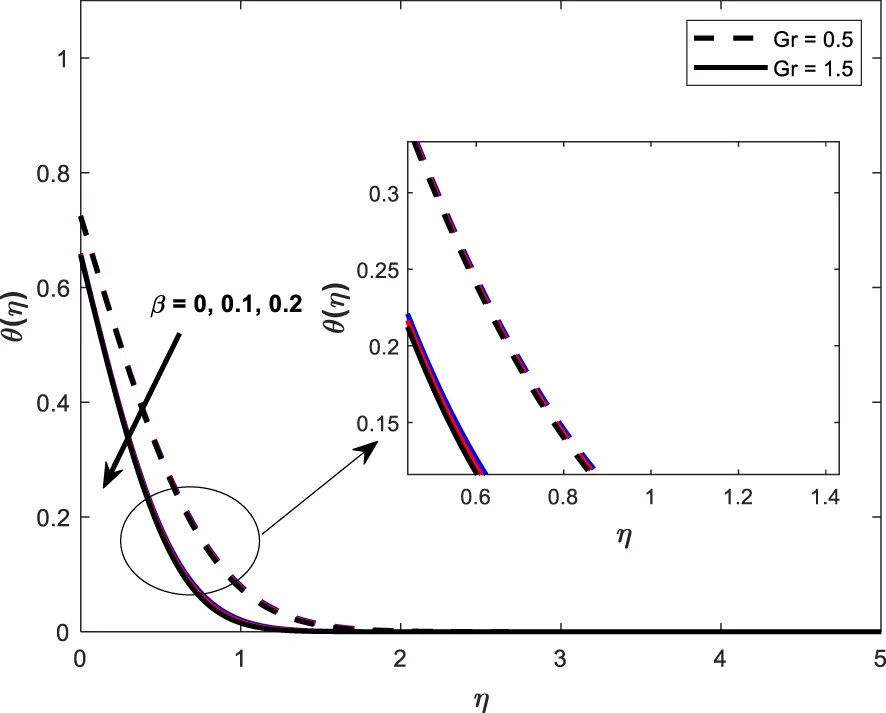
Influence of the thermal relaxation parameter β on temperature distribution.

The selected ranges *Br* = 3.0–4.0 and α_1_ = 0.1-0.3 are considered to examine the relative contributions of viscous dissipation and thermal effects to irreversibility in the blood-based hybrid nanofluid. The Brinkman number represents the relative importance of viscous heat generation compared with conductive heat transfer; therefore, its variation provides a measure of the influence of frictional heating on entropy generation. The temperature ratio parameter represents the imposed temperature difference relative to the reference fluid temperature and determines the contribution of thermal gradients to irreversibility. These values are treated as dimensionless parametric ranges for examining the transition between heat-transfer and frictional irreversibilities rather than as direct physiological measurements. In the biomedical context, these parameters provide a dimensionless assessment of how viscous heating and temperature differences can modify thermal transport in blood-based systems, while the representative blood-flow conditions discussed in the Biomedical relevance subsection provide the corresponding practical context.

Figures 13, 14 illustrate the impact of the Brinkman number on entropy generation and the Bejan number. For the considered conditions, a greater Brinkman number lowers the Bejan number and raises the entropy generation rate. In essence, the Brinkman number compares the heat transfer via molecular conduction to the heat produced by viscous heating. As the Brinkman number increases, viscous dissipation becomes stronger relative to conductive heat transfer, resulting in greater entropy generation. The corresponding decrease in the Bejan number indicates an increasing contribution of fluid-friction irreversibility. For *Gr* = 0.5, *N*_*G*_ > 0.5 near the cone indicates heat-transfer-dominated irreversibility, whereas *N*_*G*_ < 0.5 farther from the surface indicates friction-dominated irreversibility. Over the considered range *Br* = 3–4, increasing Br shifts the *N*_*G*_ = 0.5 transition toward the cone surface. For *Gr* = 1.5, this transition occurs closer to the surface than for *Gr* = 0.5.

**Figure 13:**
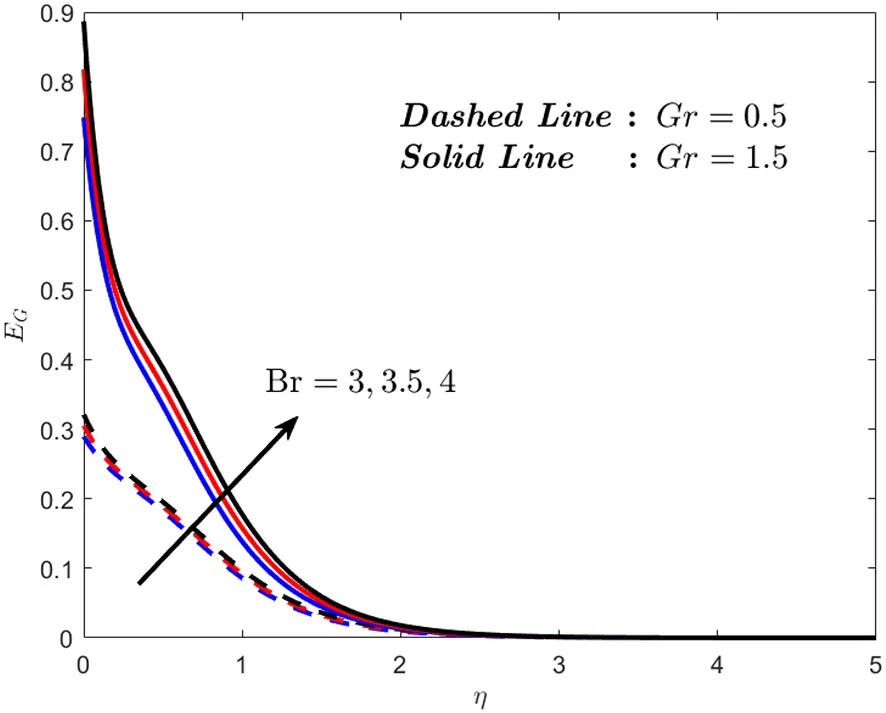
Influence of the Brinkman number Br on entropy generation.

**Figure 14:**
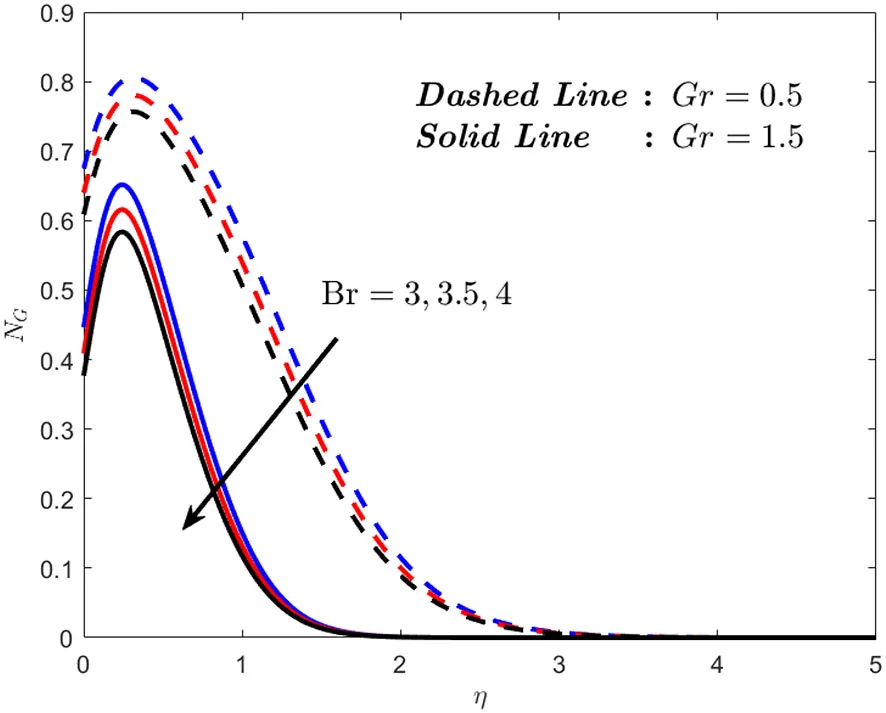
Influence of the Brinkman number Br on Bejan number.

Figures 15, 16 illustrate the effects of the temperature ratio parameter on entropy generation and Bejan number. Both quantities exhibit increasing trends for larger temperature ratio parameters. As the temperature ratio parameters rise, heat transfer becomes more critical than frictional fluid effects and magnetic effects. Consequently, entropy generation increases. As we move away from the surface, entropy generation approaches zero. For the considered range α_1_ = 0.1-0.3, the increase in the Bejan number indicates a greater contribution of heat-transfer irreversibility. The region where *N*_*G*_ > 0.5 extends farther from the cone surface as α_1_ increases, indicating that the transition to friction-dominated irreversibility occurs farther into the boundary layer. Using blood as the base fluid, Figures 17, 18 shows that the nanoparticle volume fraction of Au and Cu nanoparticles affects the skin friction coefficient and Nusselt number for different values of the Grashof number. The skin-friction coefficient increases with the nanoparticle volume fraction and Grashof number, as observed in Figure 17. Increasing the Au–Cu nanoparticle concentration raises the effective viscosity of the hybrid nanofluid, which increases the resistance to deformation and enhances the wall shear stress. Although the velocity profile decreases with increasing nanoparticle loading, the corresponding increase in effective viscosity can still result in a larger skin-friction coefficient. Increasing Gr strengthens the buoyancy force relative to viscous resistance, which intensifies near-wall momentum transport and increases the velocity gradient at the cone surface. Hence, the wall shear stress and skin-friction coefficient increase under the considered buoyancy conditions.

**Figure 15:**
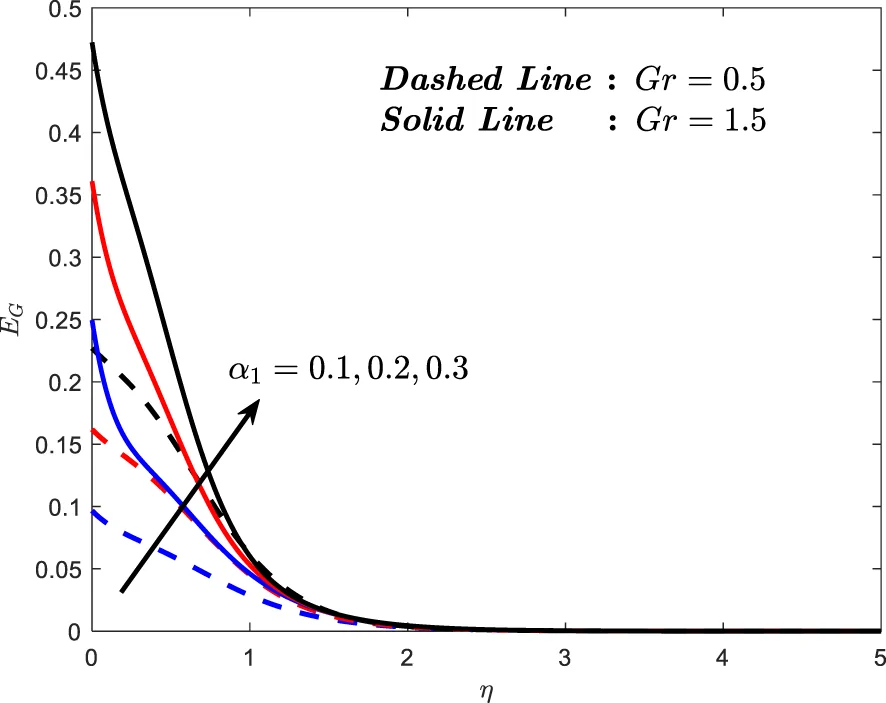
Influence of the temperature ratio parameter α_1_ on entropy generation.

**Figure 16:**
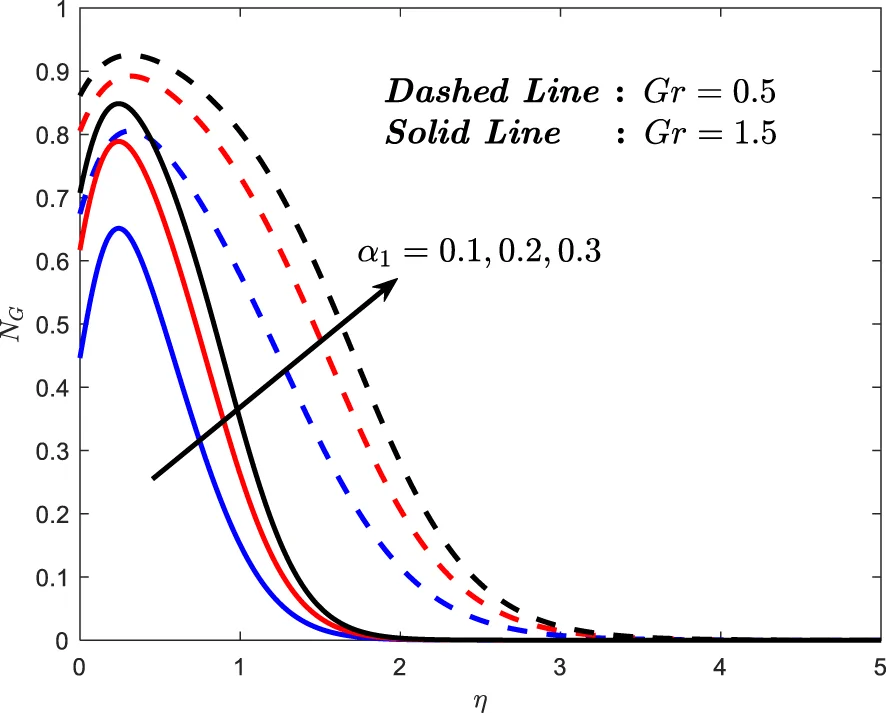
Influence of the temperature ratio parameter α_1_ on Bejan number.

**Figure 17:**
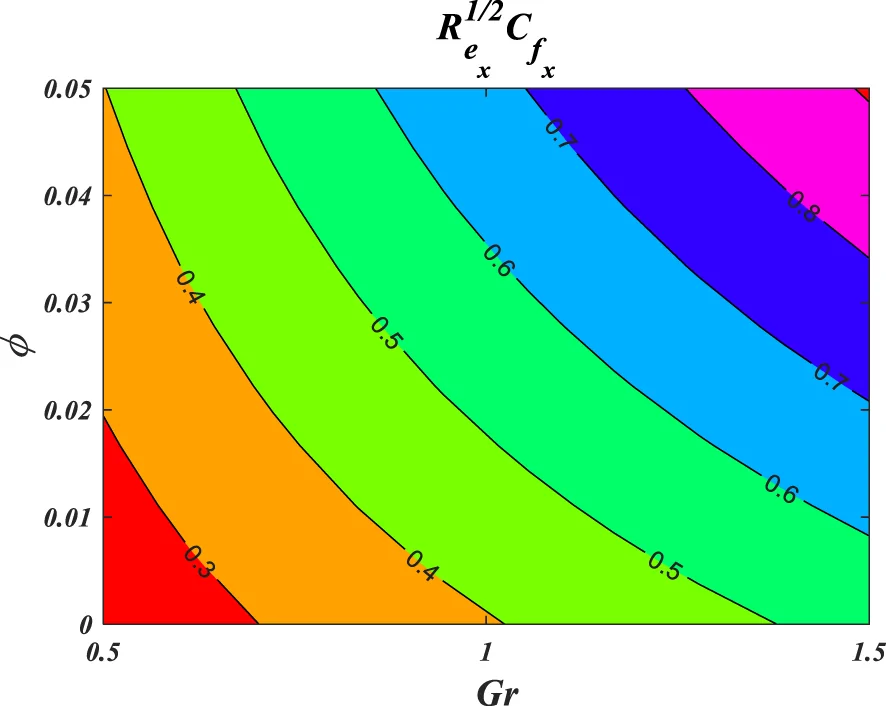
Influence of nanoparticle volume fraction ϕ and the Grashof number Gr on skin friction.

**Figure 18:**
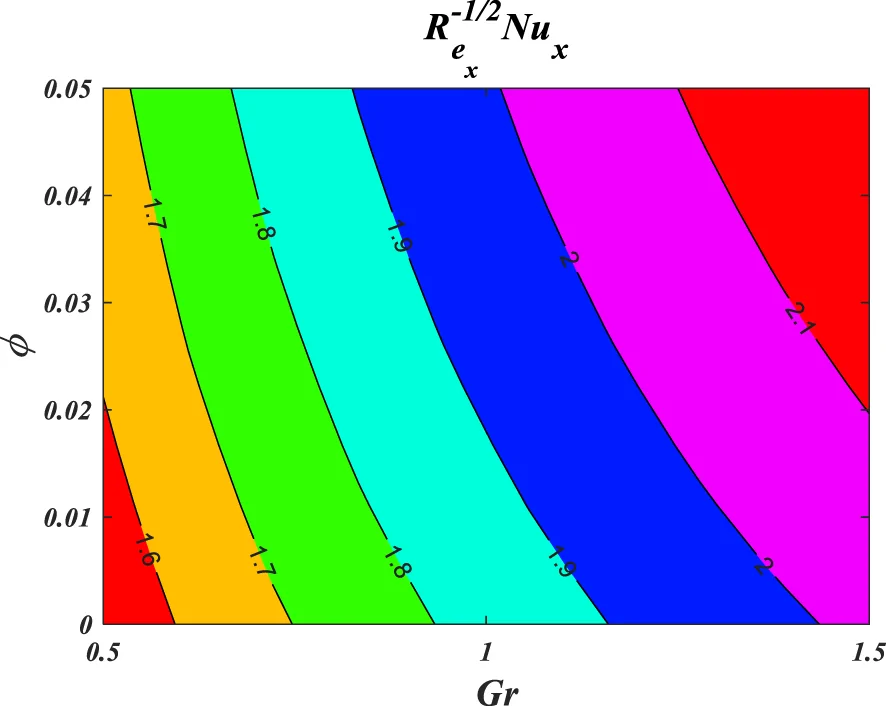
Influence of volume fraction ϕ and the Grashof number on the Nusselt number.

Figure 18 illustrates that the Nusselt number also increases with the nanoparticle volume fraction and the Grashof number. This enhancement results from the improved effective thermal conductivity and stronger buoyancy-driven convection, which together promote heat transfer. Increasing the nanoparticle volume fraction enhances the effective thermal conductivity of the hybrid nanofluid, while a higher Grashof number strengthens buoyancy-driven convection. These combined effects increase the temperature gradient at the cone surface and consequently enhance the Nusselt number. The reduction in wall resistance with increasing velocity slip weakens the velocity gradient at the cone surface, thereby reducing the skin-friction coefficient.

Figure 19 illustrates that skin friction exhibits distinct behavior. Because of the higher viscosity of the nanoparticles, it increases with the nanoparticle volume fraction; however, it decreases with the velocity slip parameter, as surface slip lessens the drag on the wall. The reduction in wall resistance with increasing velocity slip weakens the velocity gradient at the cone surface, thereby reducing the skin-friction coefficient. Figure 20 demonstrates the combined effects of the thermal radiation parameter and the nanoparticle volume fraction parameter of Au and Cu nanoparticles on the heat transfer performance of a blood-based hybrid nanofluid. When the radiation parameter and nanoparticle volume fraction increase, the Nusselt number also increases, indicating enhanced heat transfer. This enhancement is due to the increased contribution of radiative heat transfer and the improved effective thermal conductivity associated with nanoparticle loading. Figure 21 illustrates that increasing the thermal slip parameter reduces heat transfer by decreasing the surface temperature gradient, while increasing the nanoparticle volume fraction enhances the Nusselt number through the improvement in effective thermal conductivity.

**Figure 19:**
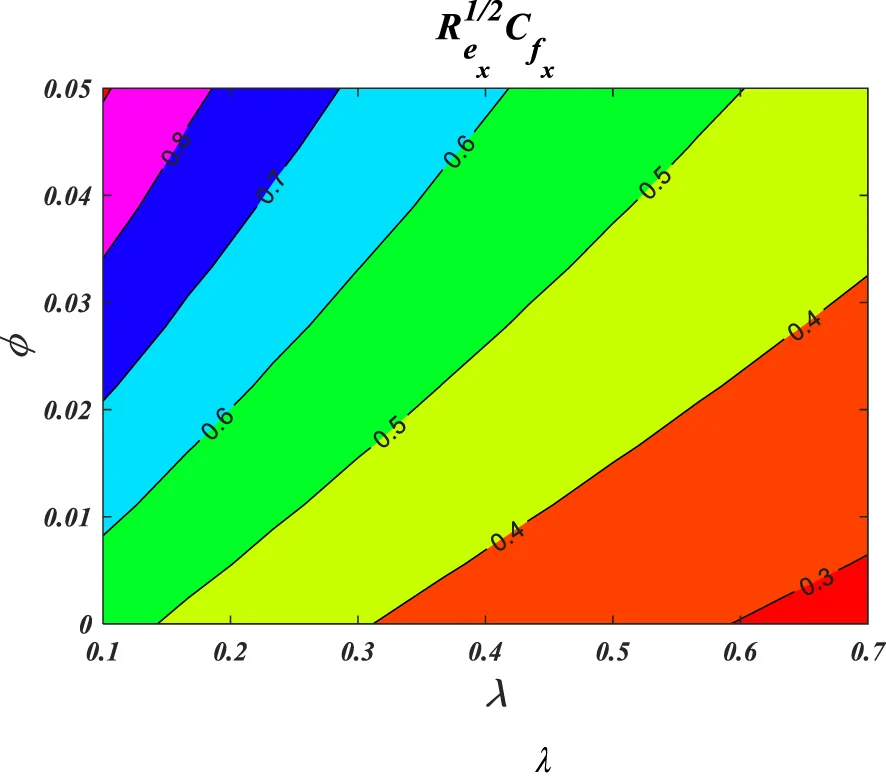
Influence of velocity slip parameter λ and nanoparticle volume fraction ϕ on Skin friction.

**Figure 20:**
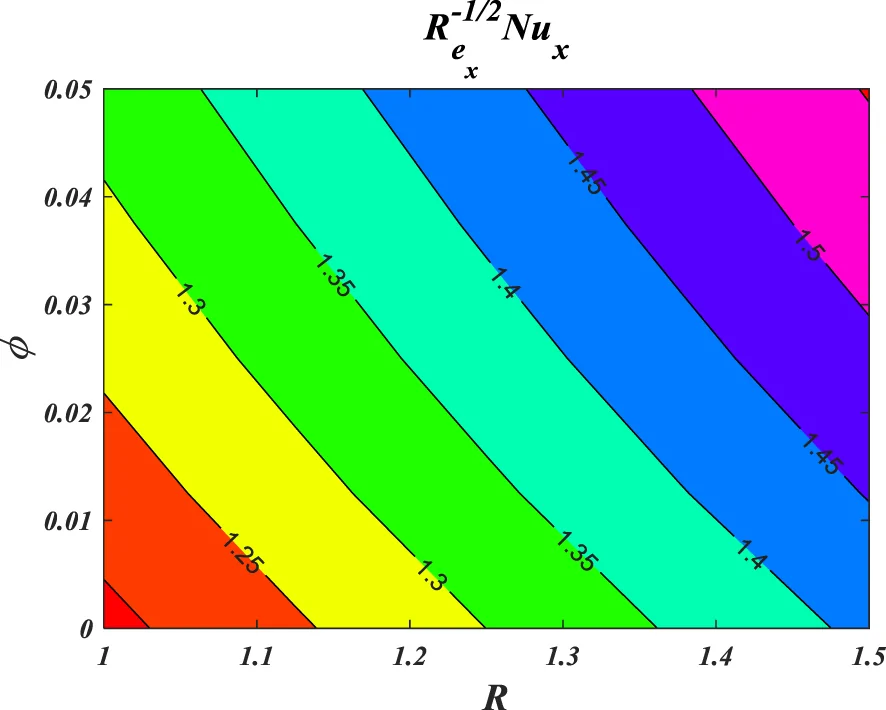
Influence of radiation parameter *R* and nanoparticle volume fraction ϕ on Nusselt number.

**Figure 21:**
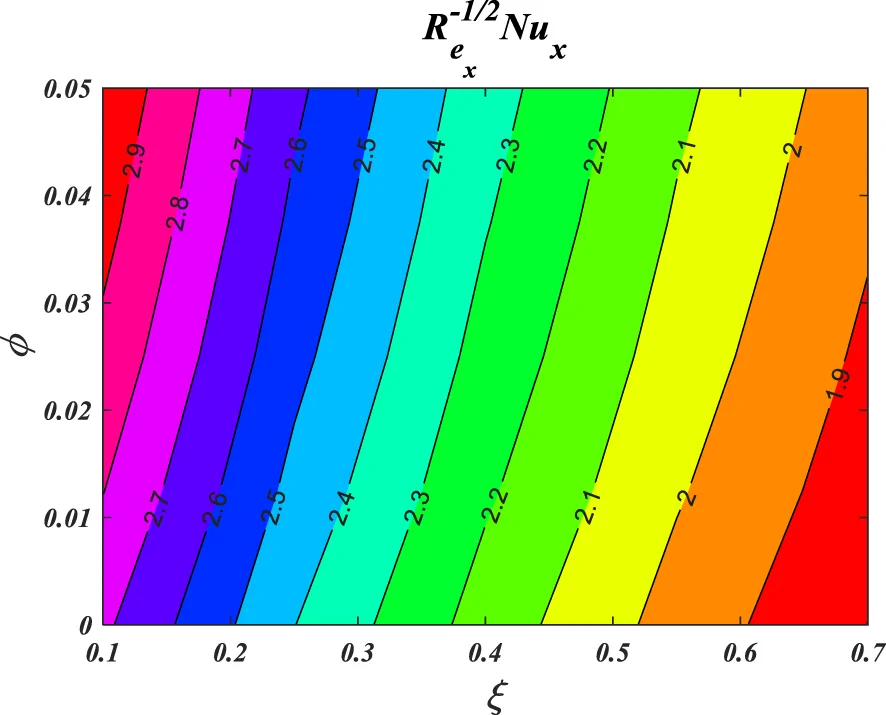
Influence of thermal slip parameter ξ and nanoparticle volume fraction ϕ on Nusselt number.

### 5.1 Biomedical relevance

The present blood-based model can be interpreted in the context of magnetically influenced biomedical transport, while recognizing the idealized nature of the vertical cone geometry. Herrera-Valencia et al. (2024) investigated human blood using a Maxwell-type viscoelastic formulation and experimental rheological data, demonstrating the importance of viscoelastic and transport mechanisms in blood flow. As a representative experimental biomedical scenario, Ligtenberg et al. (2024) investigated magnetically actuated intravascular devices in an *ex vivo* porcine aorta under controlled pulsatile blood flow. Their experiments demonstrated magnetic navigation within arterial blood flow. These findings provide a practical context for interpreting the effects of magnetic resistance, viscoelasticity, and transport mechanisms examined in the present blood-based model. However, the vertical cone represents an idealized surface rather than a specific artery; therefore, the present results provide a theoretical assessment of the underlying biomedical transport mechanisms rather than a patient-specific vascular prediction.

## 6 Sobol' sensitivity analysis

To statistically evaluate the influence of the governing parameters on the skin-friction coefficient, Nusselt number, and entropy generation at the two considered buoyancy conditions, Sobol' sensitivity analysis (Wang et al., 2023) was employed. The first-order index represents the direct contribution of an individual parameter to the output variance, whereas the total-order index accounts for both its direct influence and its interactions with the remaining parameters. The analysis was performed independently at *Gr* = 0.5 and *Gr* = 1.5. For the skin-friction coefficient and Nusselt number, the base Sobol sample size was *N* = 250, whereas *N* = 220 was used for entropy generation. Independent sampling matrices were generated with a fixed random seed of 7, and each sampled parameter combination was evaluated independently to ensure that the results were unaffected by the order of evaluation. The first- and total-order indices were evaluated using the Jansen estimators. Sampling uncertainty was quantified through 1,000 bootstrap resamples, with the 2.5th and 97.5th percentiles defining the 95% confidence intervals. A sample-size convergence analysis was also performed to assess the stability of the sensitivity rankings.

Figure 22 shows that the porosity parameter *K* is clearly dominant, accounting for roughly 71% of the output variance at *Gr* = 0.5 and 67% at *Gr* = 1.5. The next most significant parameter is the Deborah number Λ, which accounts for approximately 11% for *Gr* = 0.5 and 15% for *Gr* = 1.5. The first-order contributions of the remaining parameters are insignificant. The hierarchy of the total-order indices follows the same trend, with K remaining dominant, followed by the Deborah number and a relatively small contribution from the magnetic parameter M. The small *S*_*T*_- *S*_1_ gaps suggest that the response is governed mainly by direct effects, with modest interaction contributions.

**Figure 22:**
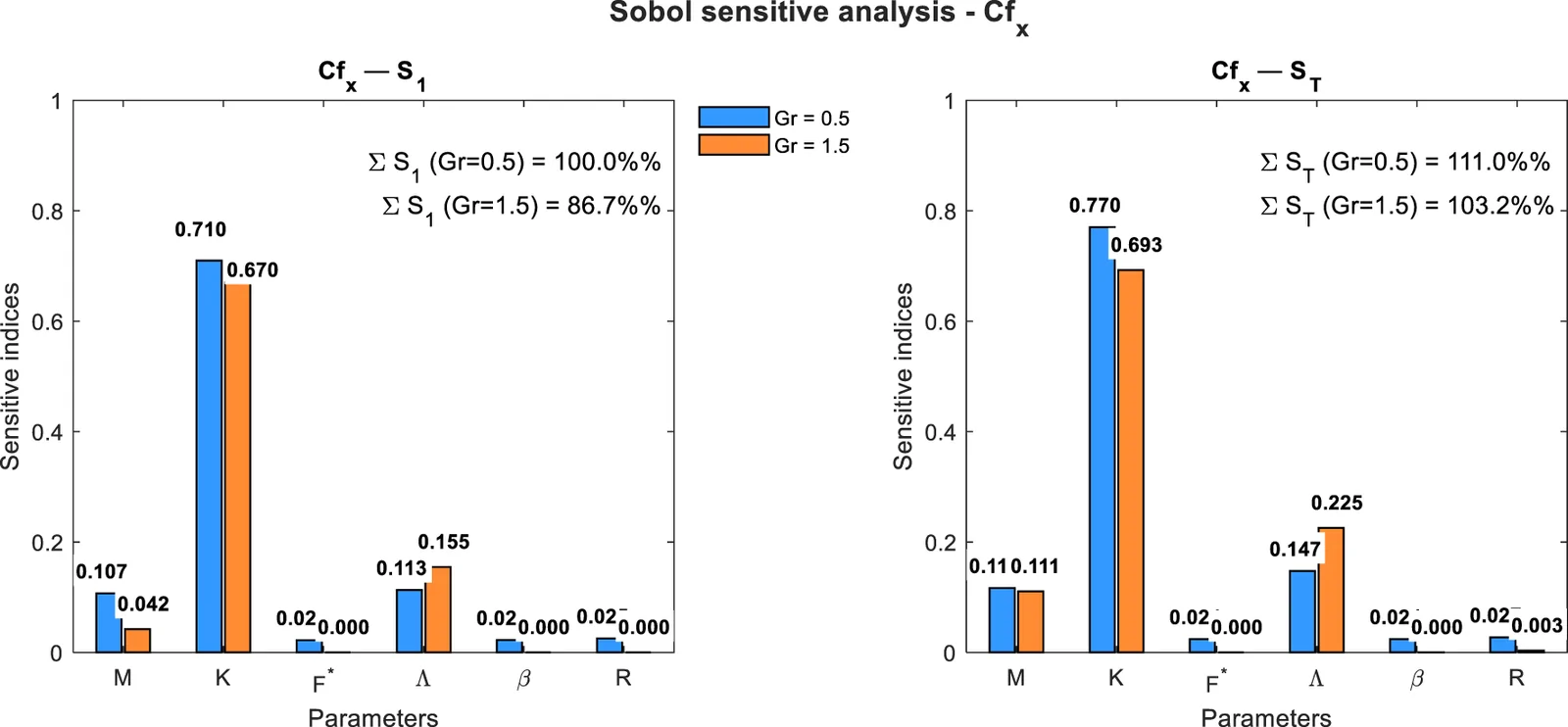
Sobol's sensitivity analysis showing the influence of model parameters on the skin-friction coefficient.

Figure 23 shows that at both buoyancy levels, the permeability parameter *K* has the dominant influence on the Nusselt number *Nu*_*x*_, explaining around 69% of the output variance at both *Gr* = 0.5 and *Gr* = 1.5. The next largest contribution is associated with the radiation parameter R, accounting for roughly 11% and 13%, whereas the magnetic parameter remains relatively small (almost 0% for *Gr* = 0.5 and 7% at *Gr* = 1.5). The total-order indices support the same overall trend. The sums of the first-order indices are 81.2% and 88.4%, indicating that the direct parameter effects account for a substantial portion of the output variance. The corresponding sums of the total-order indices are 103.8% and 101.1%; because total-order indices include overlapping interaction contributions, their sums can exceed 100%. The differences between the first- and total-order contributions indicate the presence of modest interactions. Throughout the analysis, K remains the principal controlling parameter, while R and M contribute to the coupled response.

**Figure 23:**
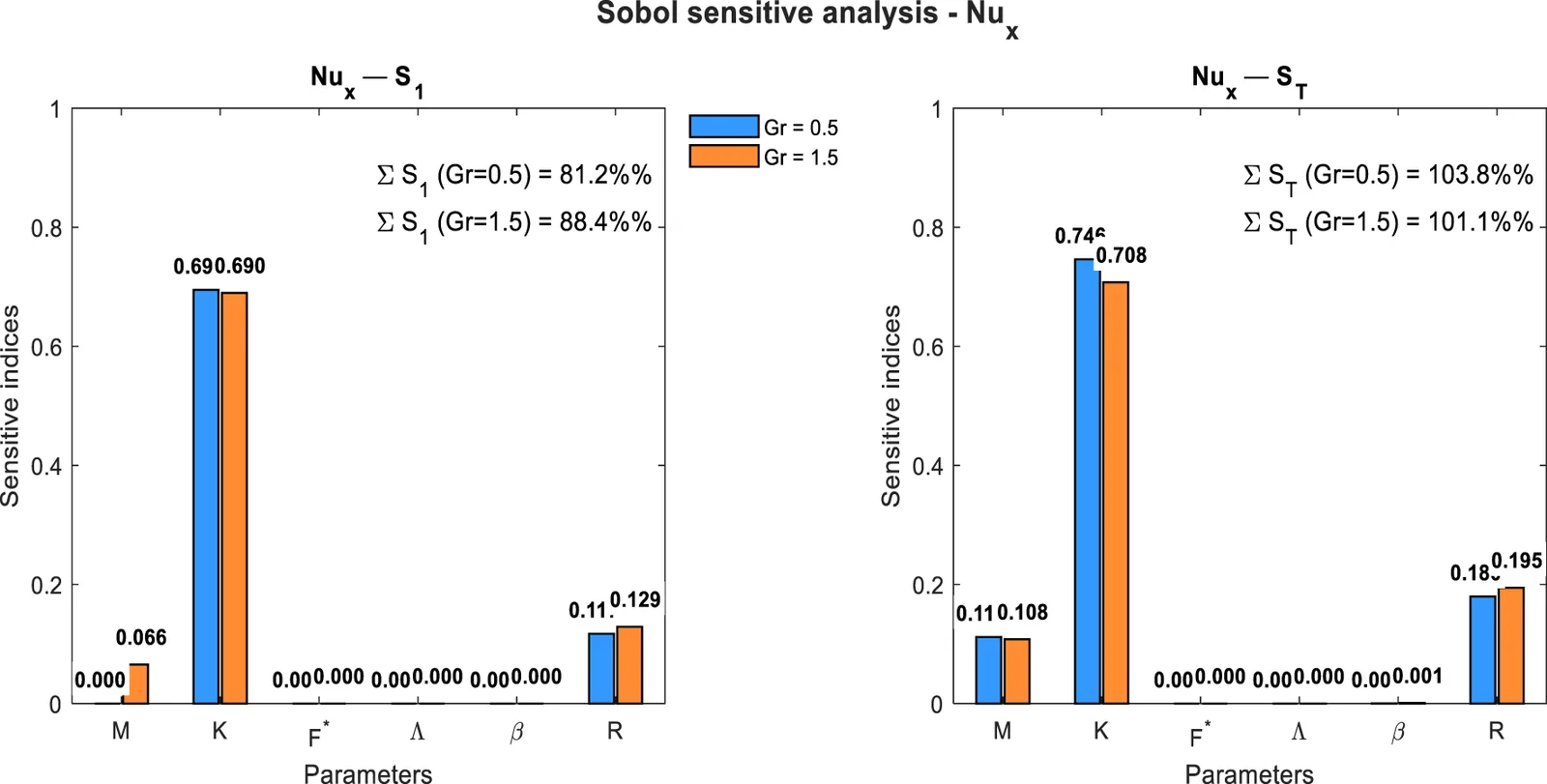
Sobol's sensitivity analysis showing the influence of model parameters on the Nusselt number.

According to Sobol' global sensitivity analysis, Figure 24 illustrates *M*, *K*, *R*, α_1_, and *Br* on the streamwise entropy generation *S*_*gen*_ at *Gr* = 0.5 and *Gr* = 1.5. Among the first-order effects, the permeability parameter K makes the largest contribution at both buoyancy levels, accounting for approximately 51.4% and 59.7%, respectively. The temperature-difference parameter α_1_ provides the next largest contribution, accounting for roughly 34.6% and 22.4%, respectively. The magnetic parameter remains relatively small, while the other weak parameters make negligible first-order contributions within the investigated ranges.

**Figure 24:**
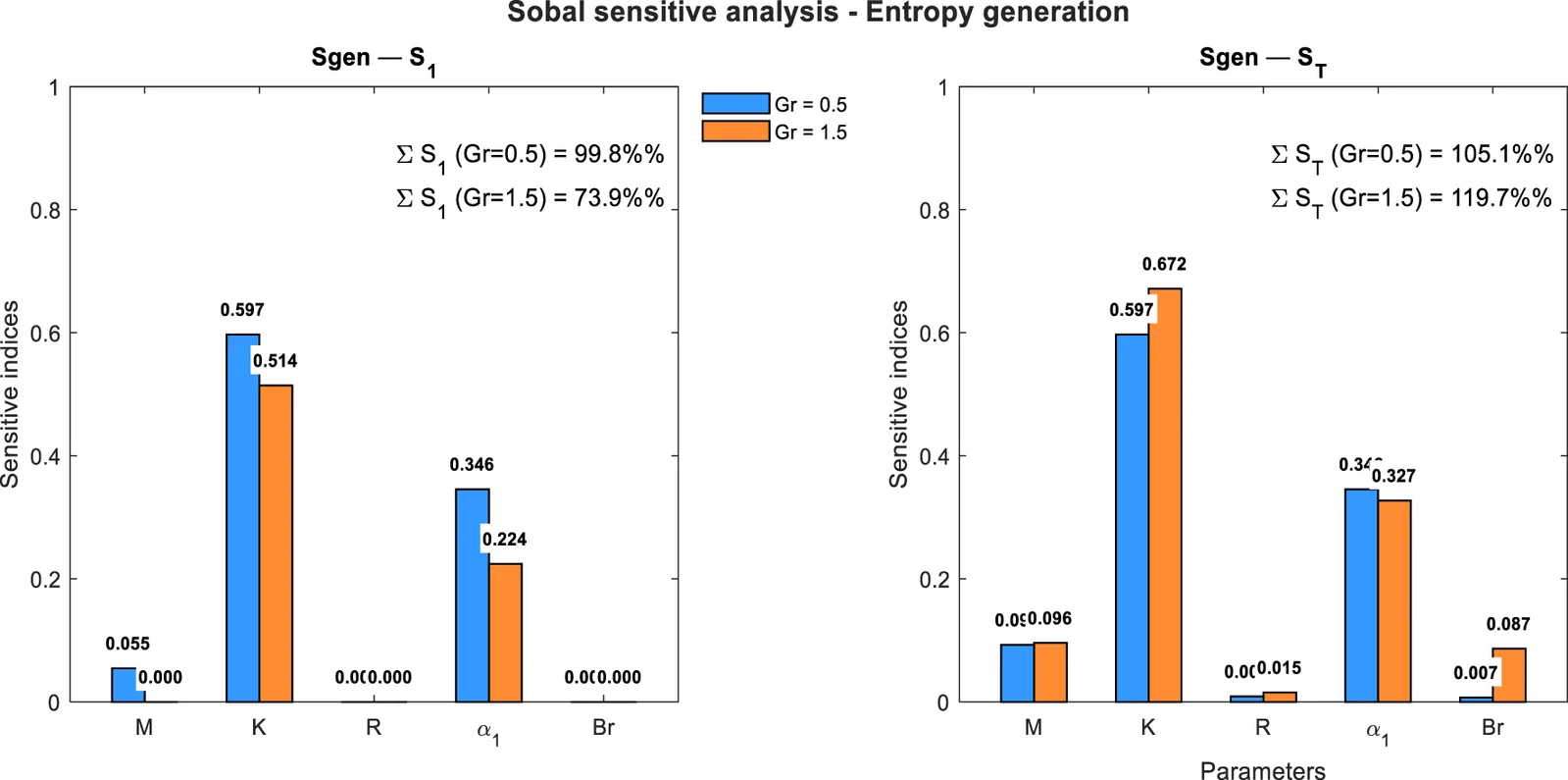
Sobol's sensitivity analysis showing the influence of model parameters on entropy generation.

The total-order results provide further information on the interaction effects. The contribution of R increases from 0.7% to 8.7% through its interactions with the other governing parameters. The overall interaction contribution increases from approximately 5% at the lower buoyancy level to about 46% at the higher buoyancy level, indicating that parameter coupling becomes more influential as buoyancy increases. Physically, this means that the irreversibility mechanisms become increasingly interdependent rather than responding solely to individual parameter variations. Controlling K remains the most effective means of regulating entropy generation, while α_1_ provides the next important control through the conductive contribution. The increasing interactions involving the secondary parameters should therefore be considered when selecting operating conditions for thermal-system optimisation, even when their individual first-order effects are small.

Figure 25 presents the 95% confidence intervals for the first- and total-order Sobol' indices. The confidence bounds support the dominant sensitivity rankings identified in Figures 22–24, particularly the strong influence of K on the skin-friction coefficient and Nusselt number and the important contributions of K and α_1_ to entropy generation. Wider confidence intervals are mainly associated with parameters having weak first-order effects, indicating greater sampling uncertainty for these small contributions. In a few cases, the first-order estimates or their confidence bounds extend slightly below zero. Such small negative estimates can arise from finite-sample uncertainty in the Sobol' estimator and are therefore interpreted as statistically negligible direct effects rather than negative physical sensitivities. Overall, the confidence analysis confirms that the principal sensitivity conclusions remain statistically consistent at both buoyancy levels.

**Figure 25:**
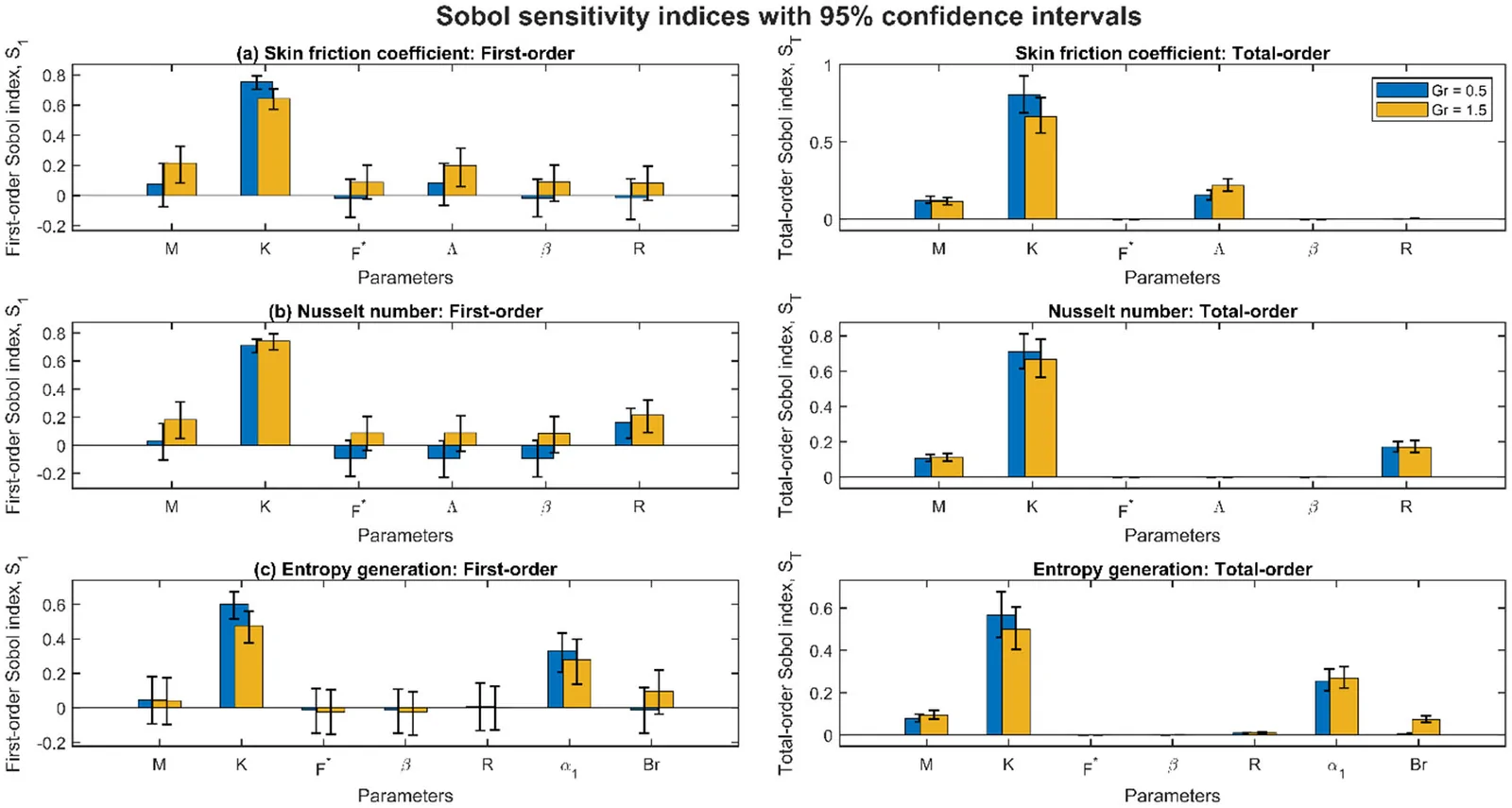
First-order (*S*_1_) and total-order (*S*_*T*_) Sobol' sensitivity indices with 95% confidence intervals for **(a)** skin-friction coefficient, **(b)** Nusselt number, and **(c)** entropy generation at the considered buoyancy levels.

Figure 26 examines the convergence of the Sobol' indices with increasing sample size. The dominant sensitivity rankings remain consistent as the sample size increases, with K retaining the leading influence on the skin-friction coefficient and Nusselt number, while K and α_1_ remain the principal contributors to entropy generation. Some fluctuations are observed for parameters with small first-order indices, particularly at lower sample sizes, but these variations do not alter the overall sensitivity ranking. The increasing stability of the dominant indices at larger sample sizes supports the robustness of the sensitivity rankings reported in the analysis.

**Figure 26:**
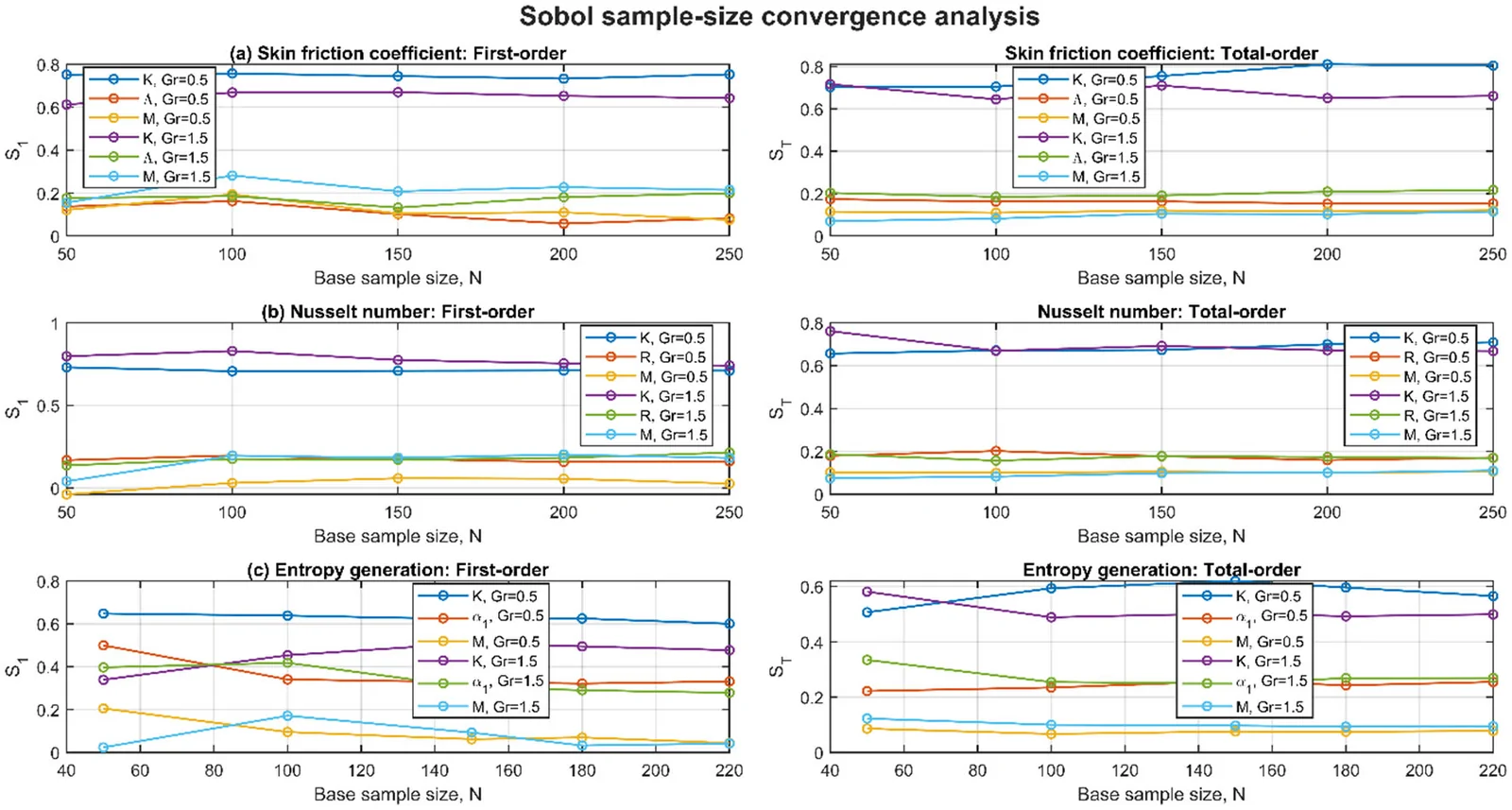
Convergence of the first-order (*S*_1_) and total-order (*S*_*T*_) Sobol' sensitivity indices with increasing sample size for **(a)** skin-friction coefficient, **(b)** Nusselt number, and **(c)** entropy generation.

## 7 ANN-based surrogate prediction

An artificial neural network (ANN) was employed as a surrogate model to predict the principal engineering responses obtained from the numerical solution of the Maxwell hybrid nanofluid flow over a cone. The ANN was developed to establish the nonlinear relationship between the governing flow and thermal parameters and the corresponding values of the skin-friction coefficient, Nusselt number, and entropy generation. The numerical solutions generated from the boundary-value model were used as reference targets for ANN training and performance assessment. Therefore, the ANN serves as a computational predictor of the numerical responses rather than an independent validation of the physical model. The overall workflow of the ANN-based prediction framework is presented in Figure 27, and the architecture of the developed ANN model is illustrated in Figure 28.

**Figure 27:**
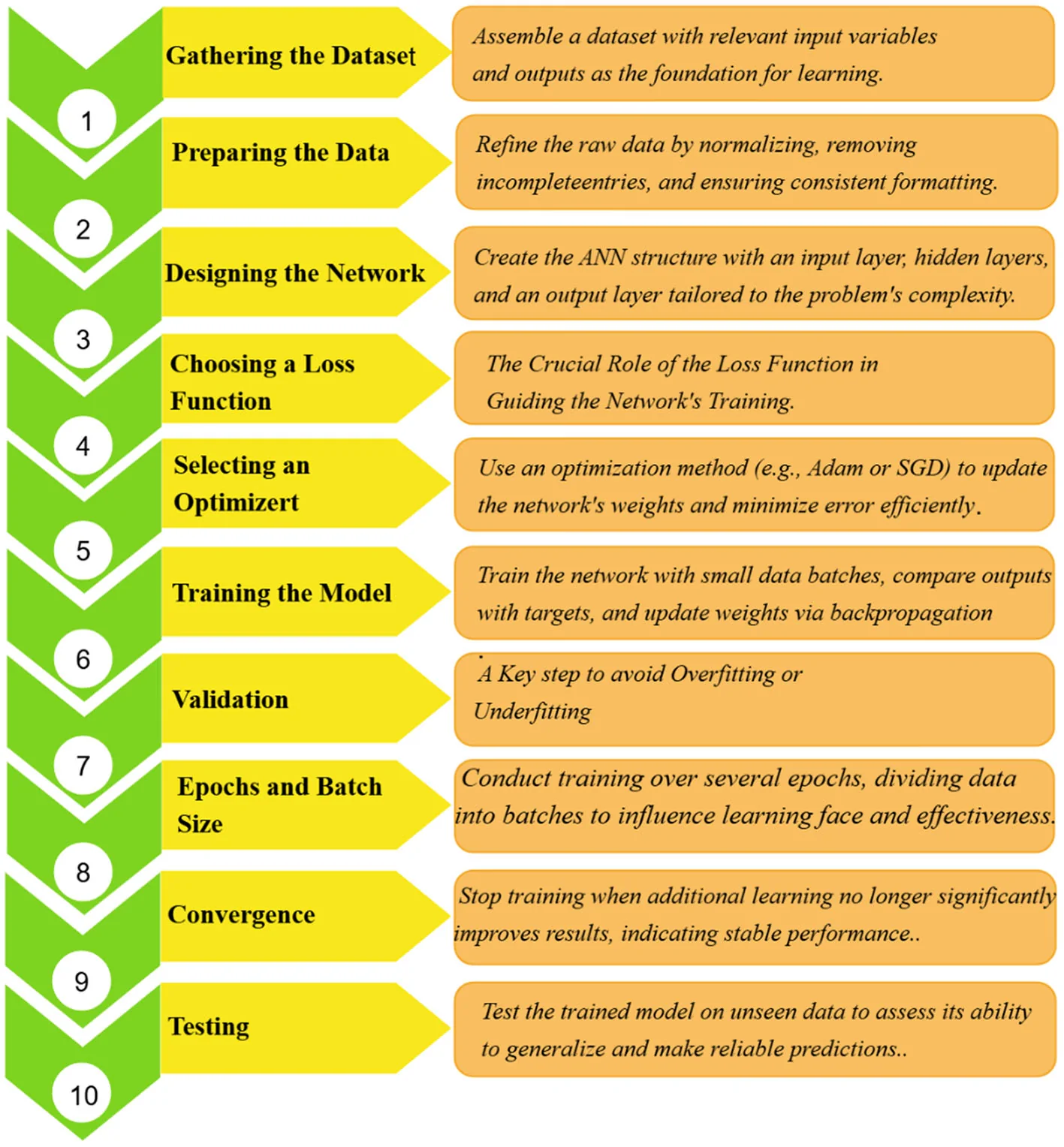
Concise workflow for training an artificial neural network (ANN).

**Figure 28:**
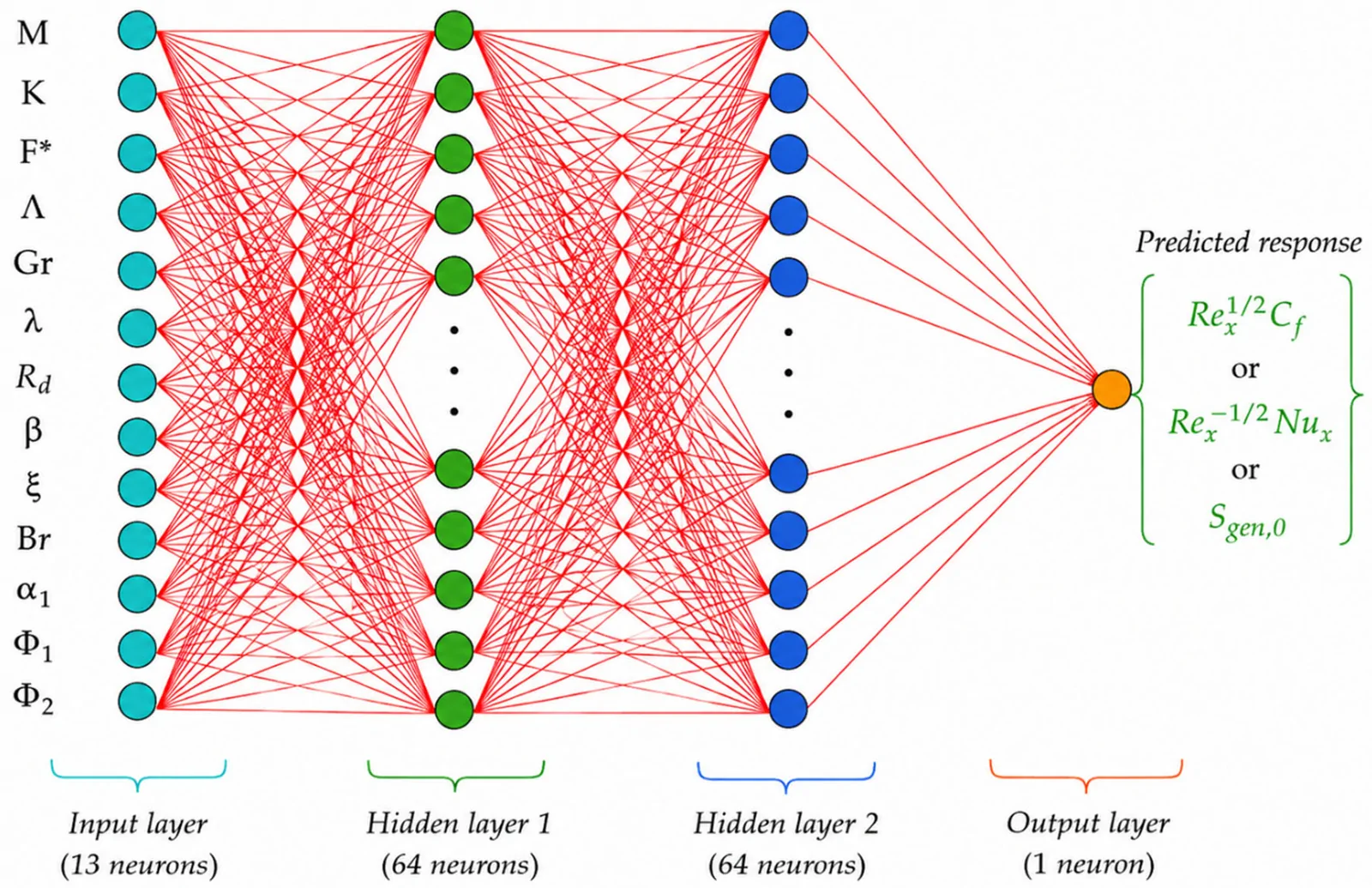
A schematic design of a multilayer ANN for the skin-friction coefficient, Nusselt number, and entropy generation.

### 7.1 ANN configuration and training procedure

The numerical dataset generated from the boundary-value solutions was used to construct the ANN models for predicting the skin-friction coefficient ${R_{e_{x}}}^{1/2}C_{f_{x}}$, Nusselt number ${R_{e_{x}}}^{-1/2}Nu_{x}$, and entropy generation *S*_*gen*, 0_. The selected governing parameters were supplied to the input layer, while each response quantity was predicted through the output layer. The data were normalized before training to reduce differences in the scales of the input variables and to support stable network learning. The ANN architecture was selected through trial evaluations of the hidden-layer configuration to obtain accurate predictions without unnecessary network complexity. The mean squared error (MSE) was employed as the loss measure, and the network weights were updated using the Adam optimization algorithm. During training, the validation data were used to monitor the predictive behavior of the network and identify the epoch associated with the minimum validation error. The independent test data were subsequently used to evaluate the ability of the trained ANN to predict previously unseen numerical samples.

### 7.2 ANN dataset generation and parameter sampling

The ANN dataset was generated from numerical solutions of the transformed boundary-value problem using independent uniform random sampling of the governing parameters. The sampling ranges were 0 ≤ *M* ≤ 2, 0.01 ≤ *K* ≤ 1, 0 ≤ F^*^ ≤ 1, 0 ≤ Λ ≤ 0.05, 0 ≤ *Gr* ≤ 2, 0 ≤ λ ≤ 1, 0 ≤ *R* ≤ 2, 0 ≤ β ≤ 0.05, 0 ≤ ξ ≤ 1, 10^−4^ ≤ *Br* ≤ 5 × 10^−3^, 1 ≤ α1 ≤ 2, 0 ≤ ϕ1 ≤ 0.02, and 0 ≤ ϕ2 ≤ 0.02. A fixed random seed was used to ensure reproducible parameter sampling. Each sampled combination was solved through a continuation-based boundary-value procedure, with an initial computational domain of 0 ≤ η ≤ 8 and adaptive domain extension when required. Parameter combinations that failed to converge or satisfy the far-field tolerance of 2 × 10^−3^ were discarded, and the sampling process was continued until 1,500 valid numerical solutions were retained. The corresponding $Re_{x}^{\frac{1}{2}}C_{f}$, $Re^{-\left(\frac{1}{2}\right)}\;Nu_{x}$, and *S*_*gen*, 0_ values were extracted from the converged solutions and used as the target responses for ANN training, validation, and testing.

### 7.3 ANN methodology and configuration

The numerical dataset contained 1,500 samples and was divided into training, validation, and testing subsets using a nominal ratio of 70:15:15. Owing to integer sample allocation, the resulting subsets contained 1,049 training samples, 226 validation samples, and 225 testing samples, corresponding to 69.9%, 15.1%, and 15.0%, respectively. The same sample indices were retained for all three ANN models to ensure a consistent basis for comparison.

Three independent single-output ANN models were developed to estimate the skin-friction response, heat-transfer response, and wall entropy-generation response. Each network contained 13 input neurons, two hidden layers with 64 neurons in each layer, and one output neuron. Rectified Linear Unit (ReLU) activation was employed in the hidden layers, whereas a linear activation was used at the output layer. The three predicted responses were ${R_{e_{x}}}^{1/2}C_{f_{x}}$, ${R_{e_{x}}}^{-1/2}Nu_{x}$, and S_gen,0_.

Before training, the input variables were standardized using the mean and standard deviation calculated only from the training subset. The corresponding target variable of each ANN was standardized in the same manner using its training data. This procedure prevents information from the validation and testing subsets from entering the scaling stage. Mean squared error (MSE) was used as the loss function, and the network weights were updated using the Adam optimizer.

Training was initiated with a learning rate of 0.001 and a batch size of 32, with a maximum limit of 500 epochs. A ReduceLROnPlateau learning-rate scheduler was used with a reduction factor of 0.5 and a patience of 30 epochs. Early stopping with a patience of 80 epochs was applied according to the validation loss, and the network state corresponding to the minimum validation loss was restored for the final predictions. A random seed of 42 was maintained to support reproducibility.

The best validation epochs were 265, 177, and 141 for ${R_{e_{x}}}^{1/2}C_{f_{x}}$,${R_{e_{x}}}^{-1/2}Nu_{x}$, and S_gen,0_, respectively. Training terminated at epochs 345, 257, and 221 after the early-stopping criterion was reached. The corresponding minimum validation MSE values during training were 0.00310818, 0.00449019, and 0.02355748, respectively. These training-loss values refer to the standardized target space; the final prediction metrics reported below were calculated after transforming the ANN outputs back to their original physical scale.


$${\tilde{x}}_{i}\;=\;\left(x_{i}\;-\;\mu_{x}\right)/\sigma_{x}$$

$${ỹ}_{i}\;=\;\left(y_{i}\;-\;\mu_{y}\right)/\sigma_{y}$$

$$MSE\;=\;\left(1/N\right)\sum_{i=1}^{N}{\left(y_{i}-\;{ŷ}_{i}\right)}^{2}$$

The ANN architecture, training configuration, and output-specific training results are summarized in Tables 7, 8, while the performance measures obtained for the training, validation, testing, and complete datasets are reported in Table 9.

**Table 7:** ANN architecture and training configuration.

ANN characteristic	Setting
Total samples	1,500
Training / validation / testing	1,049 / 226 / 225
Nominal dataset split	70% / 15% / 15%
Actual dataset split	69.9% / 15.1% / 15.0%
Number of ANN models	3 independent single-output models
Architecture	13–64–64–1
Hidden layers	2
Neurons per hidden layer	64
Hidden-layer activation	ReLU
Output layer	Linear
Loss function	Mean squared error (MSE)
Optimizer	Adam
Initial learning rate	0.001
Learning-rate scheduler	ReduceLROnPlateau
LR reduction factor	0.5
Scheduler patience	30 epochs
Minimum learning rate	1 × 10^−7^
Batch size	32
Maximum epochs	500
Early-stopping patience	80 epochs
Random seed	42

**Table 8:** Output-specific ANN training results.

Characteristic	${R_{e_{x}}}^{1/2}C_{f_{x}}$	${R_{e_{x}}}^{-1/2}Nu_{x}$	S_gen,0_
Best validation epoch	265	177	141
Early-stopping epoch	345	257	221
Best validation MSE	0.00310818	0.00449019	0.02355748

**Table 9:** ANN performance metrics on the original response scale.

Output	Dataset	MSE	RMSE	MAE	*R*	*R^2^*
${R_{e_{x}}}^{1/2}C_{f_{x}}$	Training	3.15932948 × 10^−6^	1.77745028 × 10^−3^	1.37605425 × 10^−3^	0.99998187	0.99996197
	Validation	2.86078634 × 10^−4^	1.69138592 × 10^−2^	1.12414910 × 10^−2^	0.99836228	0.99672461
	Testing	3.70674272 × 10^−4^	1.92529029 × 10^−2^	1.21805519 × 10^−2^	0.99744156	0.99479145
${R_{e_{x}}}^{-1/2}Nu_{x}$	Training	4.19505195 × 10^−5^	6.47692207 × 10^−3^	5.11977682 × 10^−3^	0.99995094	0.99978572
	Validation	9.65734129 × 10^−4^	3.10762631 × 10^−2^	2.03694571 × 10^−2^	0.99784001	0.99560940
	Testing	7.52526219 × 10^−4^	2.74322113 × 10^−2^	1.89598463 × 10^−2^	0.99846937	0.99641877
S_gen,0_	Training	2.51091056 × 10^−4^	1.58458530 × 10^−2^	1.15945963 × 10^−2^	0.99985997	0.99967605
	Validation	2.05589291 × 10^−2^	1.43383852 × 10^−1^	6.50228858 × 10^−2^	0.98918456	0.97842699
	Testing	6.77406276 × 10^−3^	8.23046946 × 10^−2^	4.80602197 × 10^−2^	0.99551809	0.99033344

To assess whether the ANN provides an advantage over a simpler surrogate, a second-order polynomial regression model was evaluated using the same 13 input parameters and the same unseen test data used for ANN assessment. The comparison was performed using the coefficient of determination (R^2^), root mean squared error (RMSE), and mean absolute error (MAE) as shown in Table 10.

**Table 10:** Comparison of ANN and second-order polynomial regression performance on the unseen test dataset.

Output	Model	*R^2^*	RMSE	MAE
${R_{e_{x}}}^{1/2}C_{f_{x}}$	ANN	0.994791	0.019253	0.012181
	Second-order polynomial regression	0.989314	0.027577	0.020739
${R_{e_{x}}}^{-1/2}Nu_{x}$	ANN	0.996419	0.027432	0.018960
	Second-order polynomial regression	0.994429	0.034214	0.024641
S_gen,0_	ANN	0.990333	0.082305	0.048060
	Second-order polynomial regression	0.971374	0.141635	0.109201

The ANN yields higher R^2^ values and lower RMSE and MAE values than the second-order polynomial regression model for all three predicted responses. The improvement is particularly evident for entropy generation, for which the ANN gives *R*^2^ = 0.990333, RMSE = 0.082305, and MAE = 0.048060, compared with *R*^2^ = 0.971374, RMSE = 0.141635, and MAE = 0.109201 for polynomial regression. These results show that the ANN captures the nonlinear input-output relationships more effectively than the simpler polynomial surrogate while retaining strong predictive performance on unseen data.

To further assess the reliability of the predictions, an uncertainty analysis was performed for both the numerical and ANN results. Table 11 summarizes the corresponding uncertainty measures.

**Table 11:** Uncertainty assessment of the numerical and ANN predictions for the engineering quantities.

Output	Numerical uncertainty (%)	ANN mean *R^2^*	95% CI of *R^2^*	ANN mean RMSE	95% CI of RMSE	ANN mean MAE
${R_{e_{x}}}^{1/2}C_{f_{x}}$	0.01684	0.996166	0.995727–0.996606	0.016468	0.015505–0.017431	0.011582
${R_{e_{x}}}^{-1/2}Nu_{x}$	0.02268	0.996916	0.996597–0.997234	0.025404	0.024158–0.026650	0.018660

Table 11 summarizes the uncertainty of the numerical and ANN results. The numerical uncertainty is only **0.01684%** for ${R_{e_{x}}}^{1/2}C_{f_{x}}$ and **0.02268%** for ${R_{e_{x}}}^{-1/2}Nu_{x}$, confirming stable BVP solutions under further numerical refinement. The ANN also shows consistent predictions across 10 independent runs, with mean R^2^ values above **0.996** and low RMSE and MAE values. These results support the numerical stability and predictive reliability of both approaches.

#### 7.3.1 Performance measures


$$MSE\;=\;\left(1/N\right)\sum_{i=1}^{N}{\left(y_{i}-\;{ŷ}_{i}\right)}^{2}\;,\;RMSE\;=\;\sqrt{\left(1/N\right)\sum_{i=1}^{N}{\left(y_{i}-\;{ŷ}_{i}\right)}^{2}}$$

$$MAE\;=\;\left(1/N\right)\sum_{i=1}^{N}|y_{i}\;-\;{ŷ}_{i}|$$

$$R^{2}\;=\;1\;-\;\left[\sum_{i=1}^{N}{\left(y_{i}-\;y_{i}\right)}^{2}/\sum_{i=1}^{N}{\left(y_{i}-\;ȳ\right)}^{2}\right]$$

The testing subset was not involved in network optimization or model selection and was used only after the best validation state had been restored. Consequently, the testing statistics provide an independent measure of the predictive capability of each trained ANN.

### 7.4 ANN results and discussion

The predictive capability of the developed ANN models was evaluated for the reduced skin-friction coefficient, reduced Nusselt number, and entropy generation by comparing the predictions with the corresponding numerical results. Model performance was examined using training behavior, error distributions, training-state characteristics, and regression analysis.

Figure 29 shows a sharp decrease in the training and validation MSE during the initial epochs, followed by a more gradual improvement as training progresses. The best validation performance is attained at epoch 265, while the training error continues to decrease thereafter. The validation error remains nearly stable without a sustained increase, indicating that the network retains its predictive capability beyond the training samples. The model corresponding to epoch 265 is therefore selected as the best-performing network for predicting ${R_{e_{x}}}^{1/2}C_{f_{x}}$.

**Figure 29:**
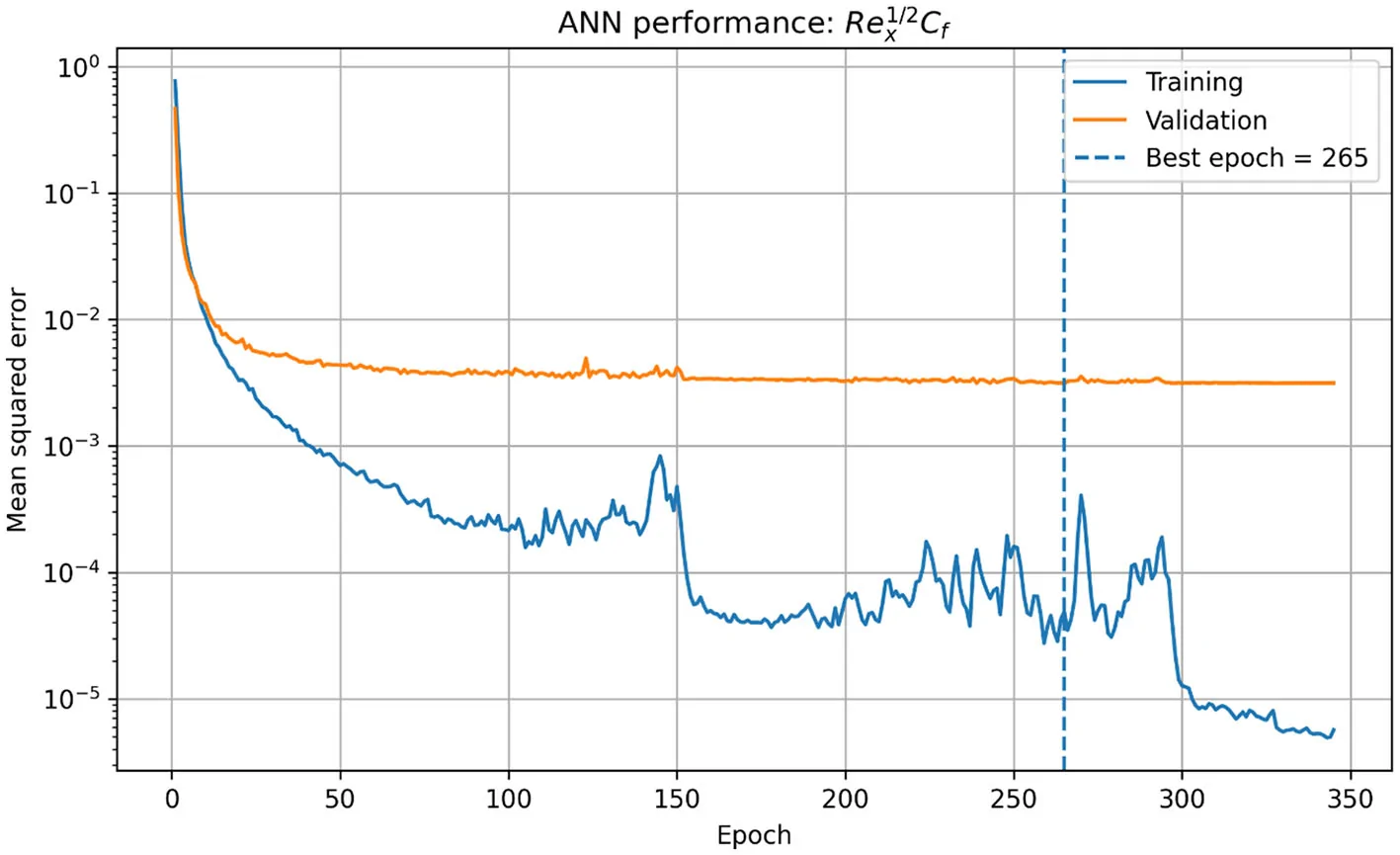
Training performance of the ANN model for the skin-friction coefficient, showing the variation of mean squared error (MSE) with epochs.

Figure 30 shows the error distribution of the ANN predictions for ${R_{e_{x}}}^{1/2}C_{f_{x}}$ across the training, validation, and testing datasets. Most residuals are concentrated close to the zero-error line, showing that the ANN predictions closely reproduce the corresponding numerical values for the majority of samples. Although a small number of errors extend farther from the central region, their occurrence is limited compared with the pronounced concentration around zero. The similar error distribution across the three data subsets, together with the dominant near-zero errors, supports the predictive accuracy and generalization of the ANN model. Figure 31 presents the regression analysis of the ANN predictions against the corresponding numerical results for ${R_{e_{x}}}^{1/2}C_{f_{x}}$. The predicted points remain close to the ideal line *Y* = *T*, while the best-fit line nearly overlaps the reference line across all data partitions. The correlation coefficients are *R* = 0.99998, 0.99836, *and* 0.99744 for the training, validation, and testing datasets, respectively, with an overall value of *R* = 0.99939. The corresponding *R*^2^ *values are* 0.99996, 0.99672, 0.99479, and 0.99877 for the training, validation, testing, and the complete dataset, respectively. These values, together with the close alignment of the predicted and numerical results, demonstrate that the ANN captures the skin-friction response with high predictive accuracy and maintains strong generalization on unseen data.

**Figure 30:**
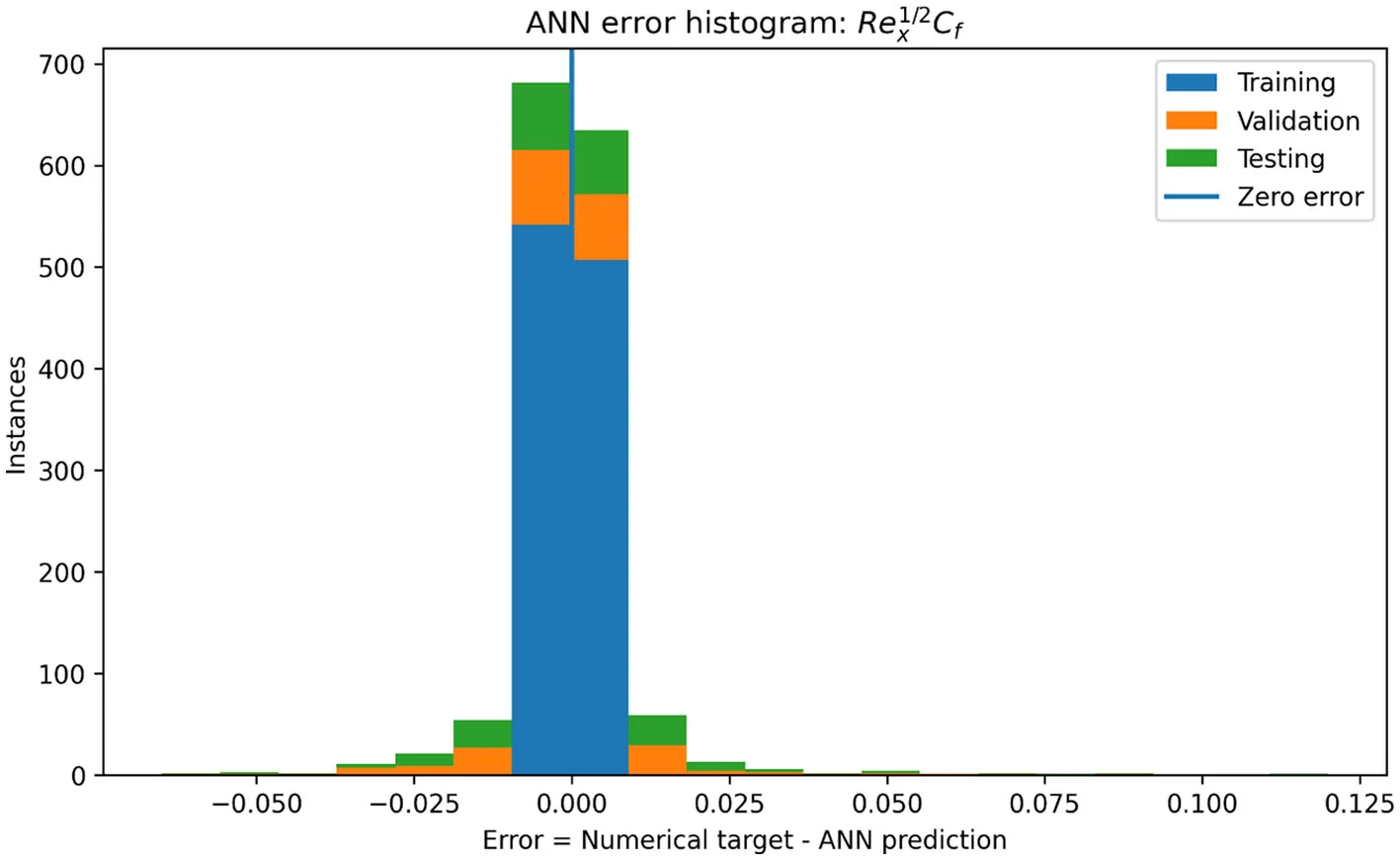
The error histogram on ${R_{e_{x}}}^{1/2}C_{f_{x}}$.

**Figure 31:**
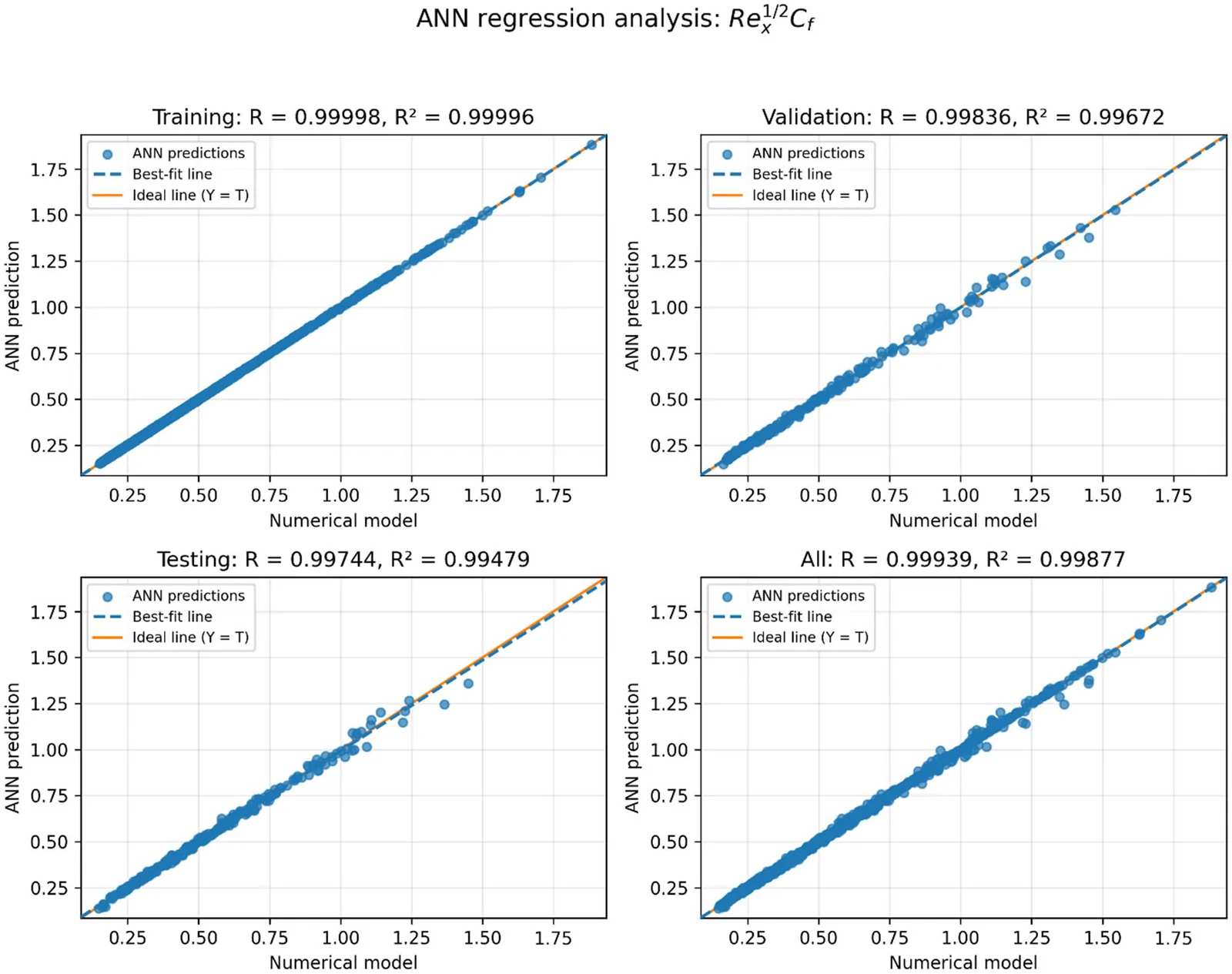
ANN regression analysis for the skin-friction coefficient across the training, validation, testing, and overall datasets.

Figure 32 presents the ANN performance for predicting ${R_{e_{x}}}^{-1/2}Nu_{x}$. The training and validation MSE decrease rapidly during the initial epochs and then improve more gradually as the learning proceeds. The minimum validation error is obtained at epoch 177, after which the validation curve remains nearly unchanged despite the continued reduction in the training error. This behavior indicates that further training mainly improves the fit to the training samples without producing a corresponding improvement in validation performance. Therefore, the network parameters associated with epoch 177 are retained as the best model for predicting the Nusselt number, thereby supporting reliable performance on unseen data. Figure 33 presents the error distribution of the ANN predictions for ${R_{e_{x}}}^{-1/2}Nu_{x}$. Most of the prediction errors for the training, validation, and testing datasets are concentrated near the zero-error line, while only a small number of samples occur farther from the central region. The concentration of residuals around zero indicates close agreement between the numerical targets and ANN predictions. The distribution on both sides of the zero-error line also suggests that the prediction errors are not dominated by a consistent overprediction or underprediction. Thus, the error pattern supports the predictive accuracy of the ANN for the Nusselt number across all three data partitions. Figure 34 presents the regression analysis of the ANN predictions against the numerical results for ${R_{e_{x}}}^{-1/2}Nu_{x}$. The ANN predictions remain closely distributed around the ideal line *Y* = *T* and the best-fit line nearly coincides with the reference line for all data partitions. The correlation coefficients are *R* = 0.99995, 0.99784, and 0.99847 for the training, validation, and testing datasets, respectively, while the complete dataset gives *R* = 0.99935. The corresponding values are *R*^2^ *values are* 0.99979, 0.99561, 0.99642, and 0.99857. The consistently high regression measures and the close agreement between the predicted and numerical values demonstrate that the ANN accurately represents the Nusselt-number response and retains strong predictive capability for unseen samples.

**Figure 32:**
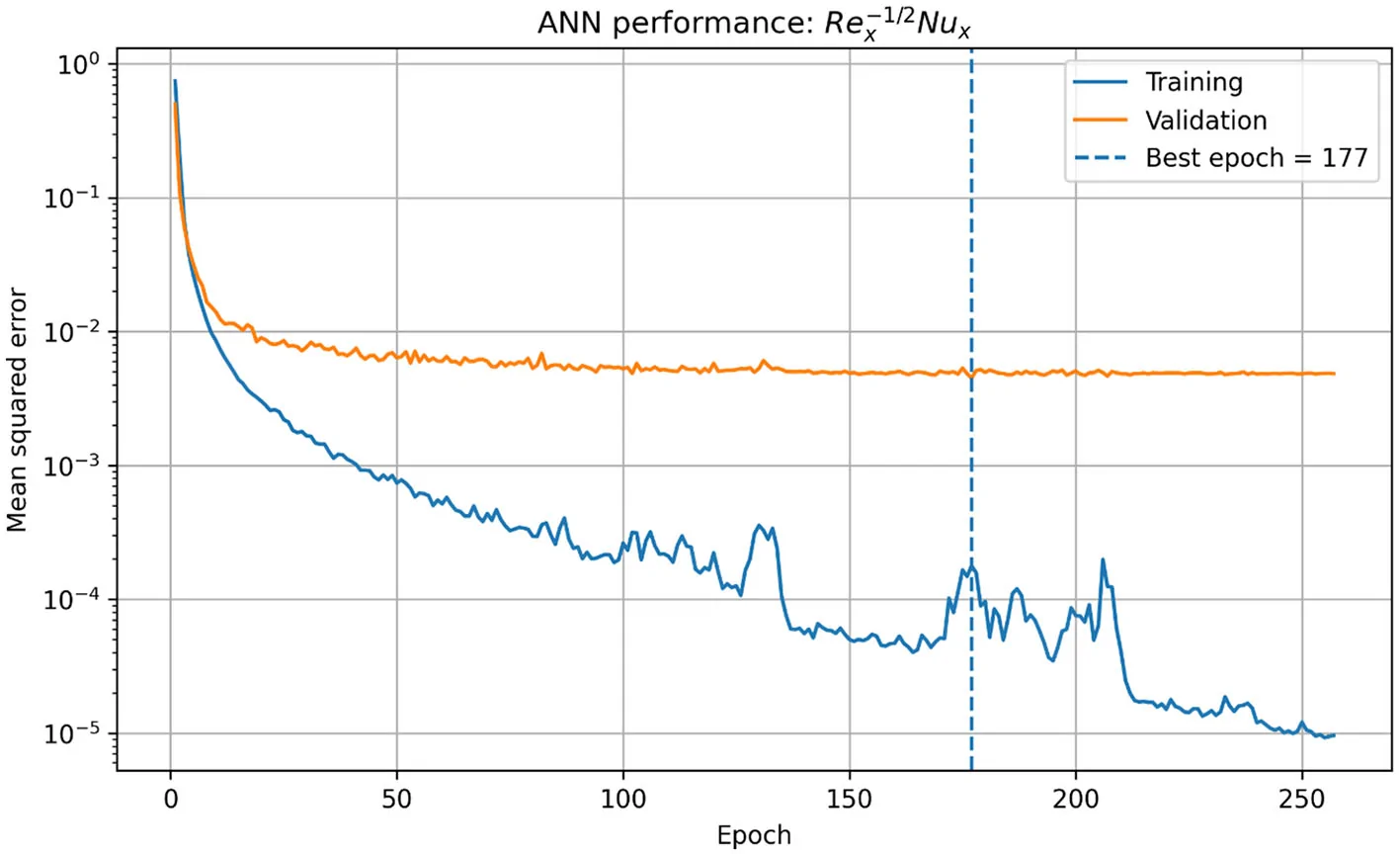
Training performance of the ANN model for the Nusselt number, showing the variation of mean squared error (MSE) with epochs.

**Figure 33:**
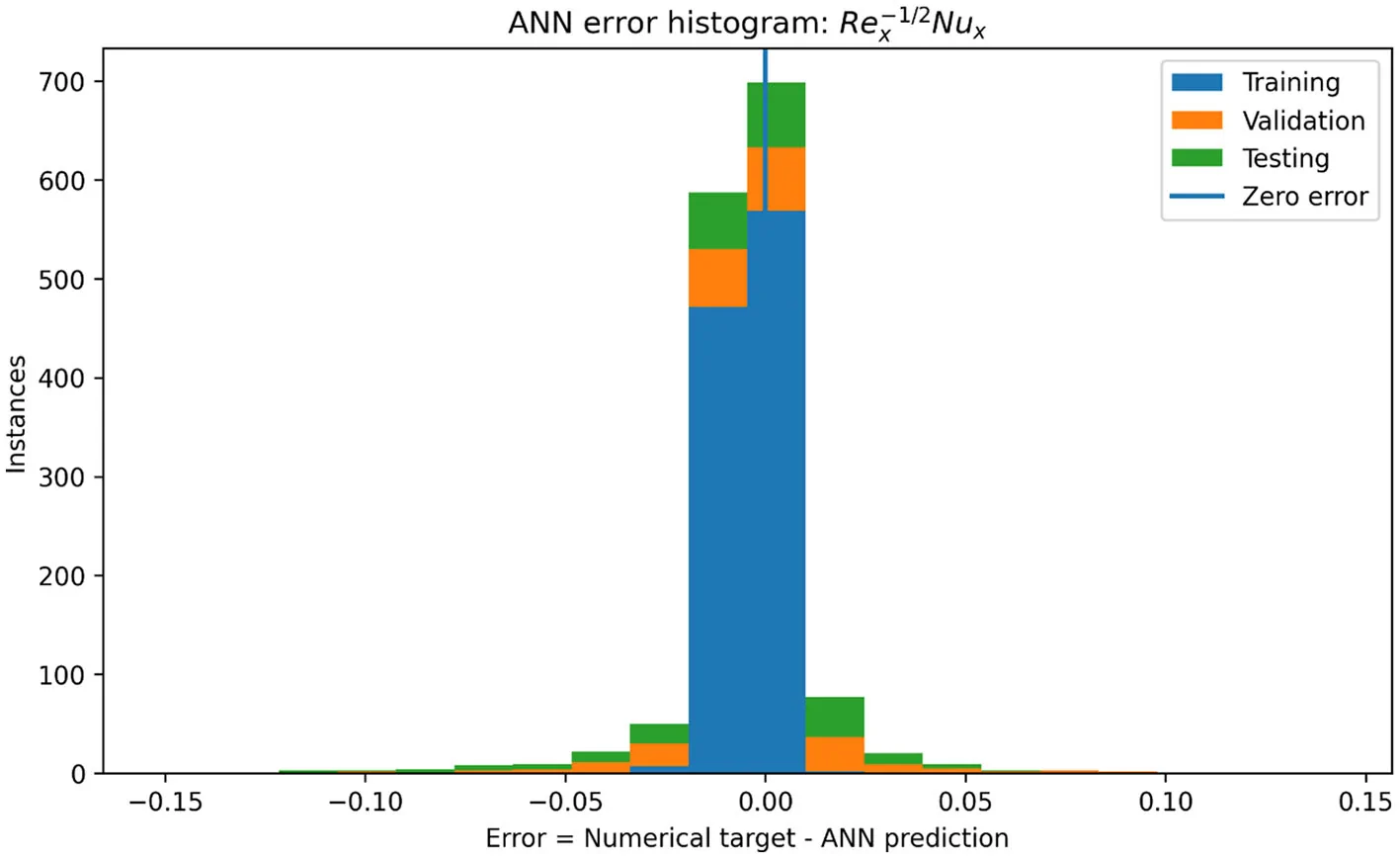
Error histogram of ANN predictions for the Nusselt number across the training, validation, and testing datasets.

**Figure 34:**
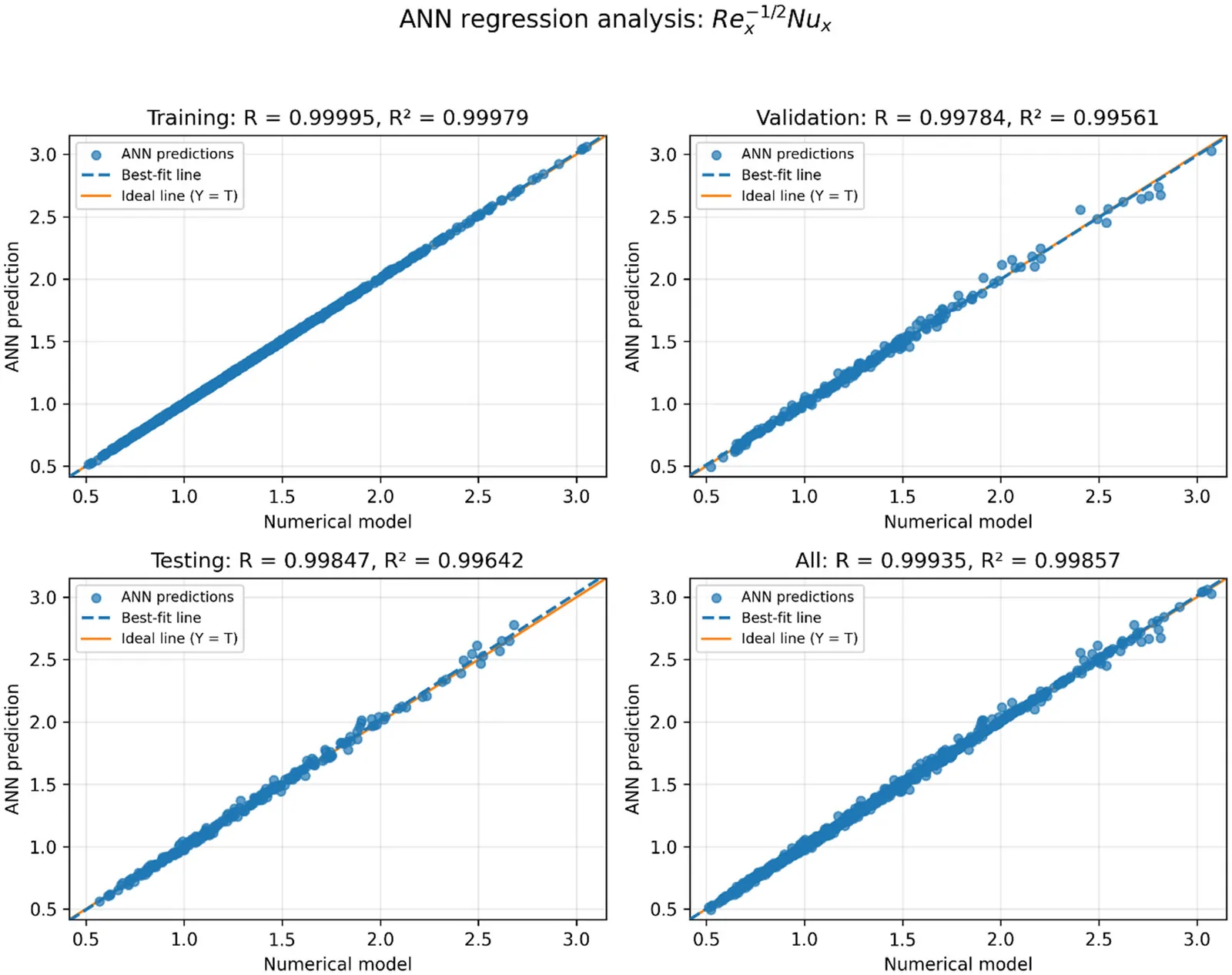
ANN regression analysis for the Nusselt number across the training, validation, testing, and overall datasets.

Figure 35 presents the ANN performance for predicting S_gen,0_. The training and validation MSE decrease sharply during the initial epochs, showing rapid learning of the entropy-generation response. The validation error subsequently approaches a nearly stable level, with the best validation performance obtained at epoch 141. In contrast, the training error continues to decrease with some intermediate fluctuations. The selection of the network state corresponding to the minimum validation error prevents the continuously decreasing training loss from being interpreted as improved generalization. These results indicate that the ANN captures the underlying entropy-generation behavior while the validation criterion provides an appropriate basis for selecting the final predictive model. Figure 36 shows that most residuals are concentrated close to the zero-error line for the training, validation, and testing datasets, indicating close agreement between the numerical targets and ANN predictions. Although a few larger deviations occur away from the central region, they represent only a small number of samples. The dominant concentration around zero indicates good predictive accuracy with no evident systematic bias in the ANN prediction of entropy generation. Figure 37 shows close agreement between the ANN predictions and the corresponding numerical targets for the training, validation, testing, and complete datasets. The regression coefficients are *R* = 0.99986, 0.98918, 0.99552, *and* 0. 99730 respectively. The predicted points remain predominantly close to the ideal line, confirming that the ANN accurately captures the entropy-generation behavior of the numerical model. Despite the high regression values, overfitting was controlled through separate validation and testing datasets and early stopping. The high test values, low prediction errors, and near-zero residuals confirm that the ANN maintains good generalization to unseen data.

**Figure 35:**
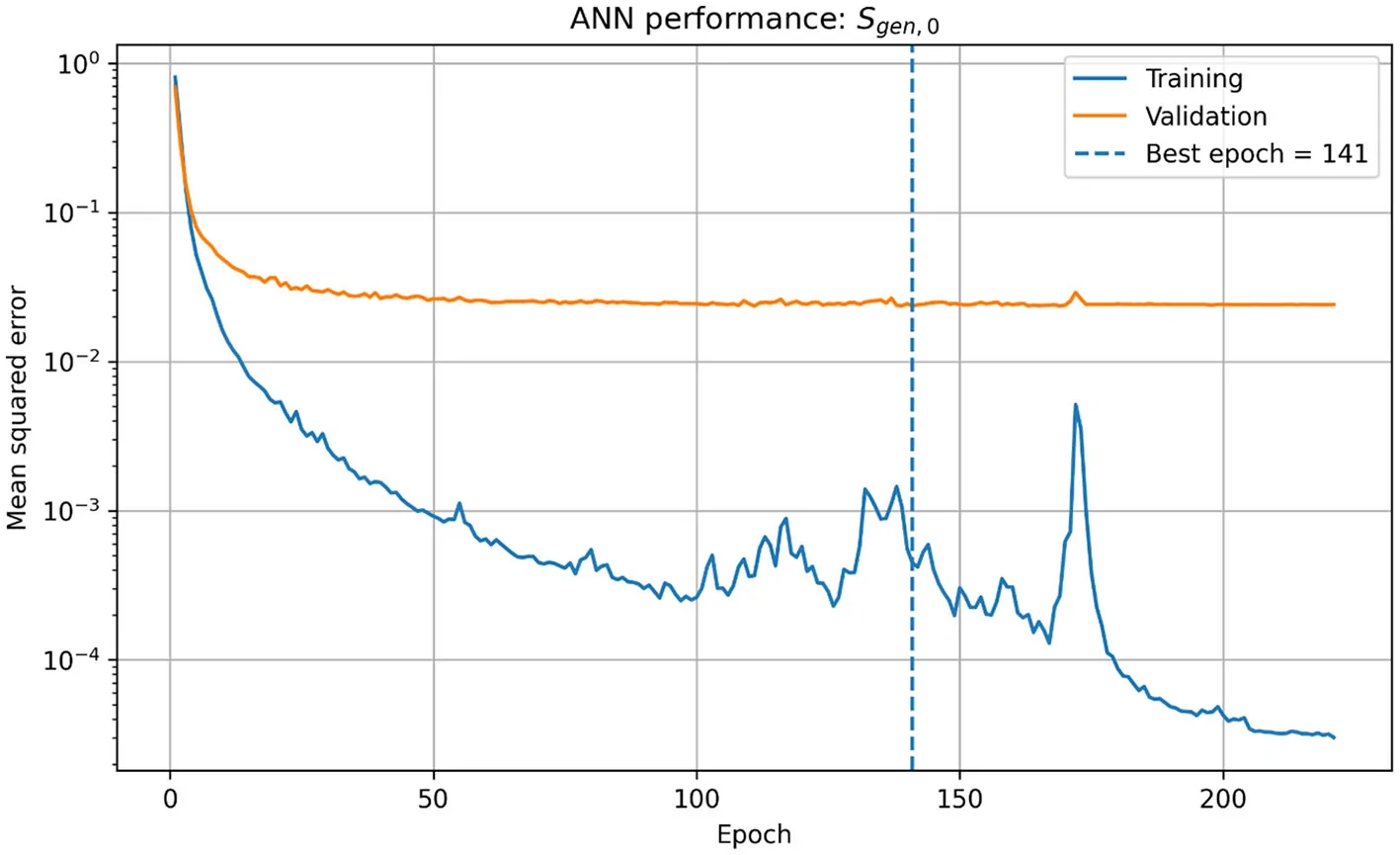
Training performance of the ANN model for entropy generation, showing the variation of mean squared error (MSE) with epochs.

**Figure 36:**
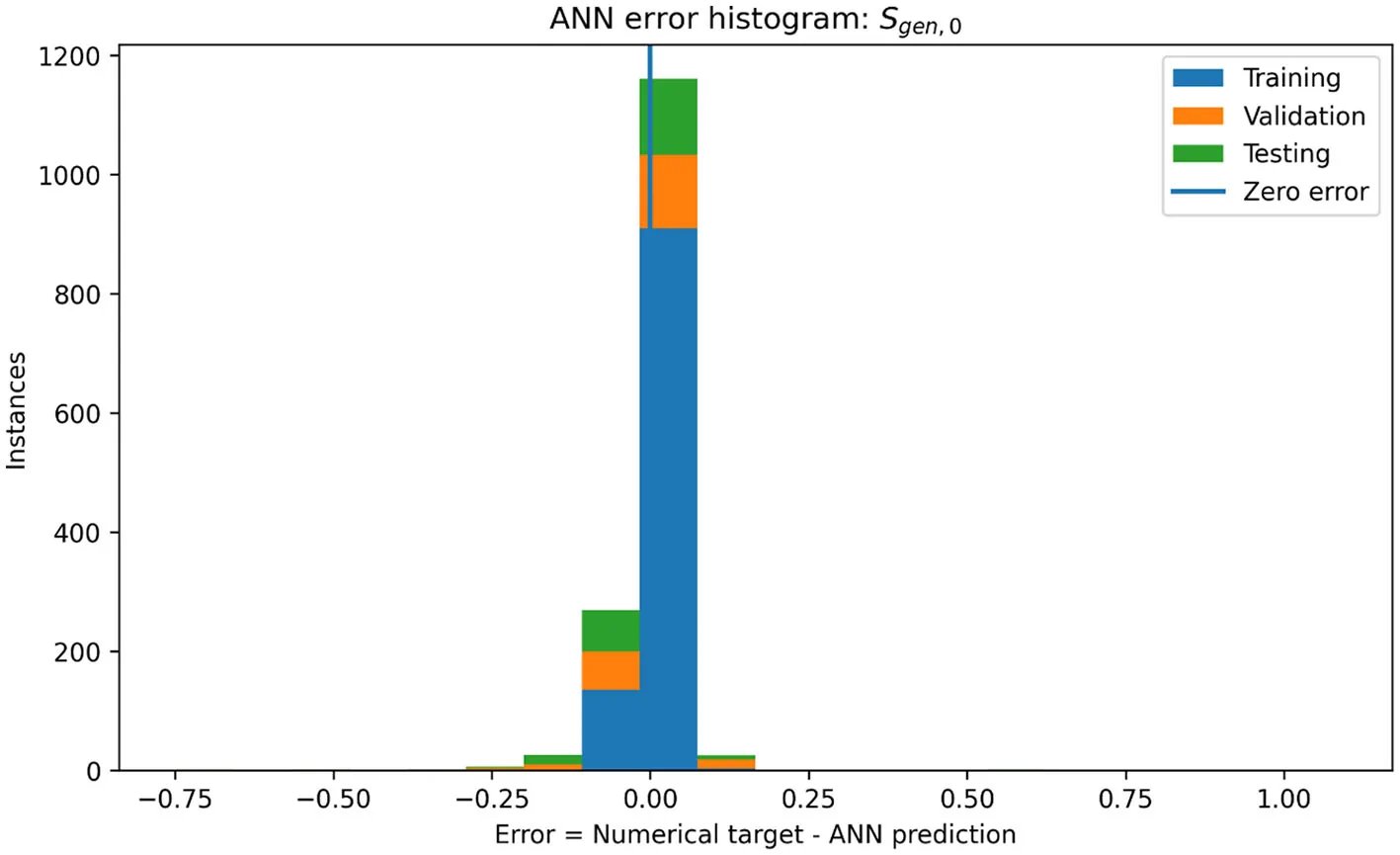
Error histogram of ANN predictions for entropy generation across the training, validation, and testing datasets.

**Figure 37:**
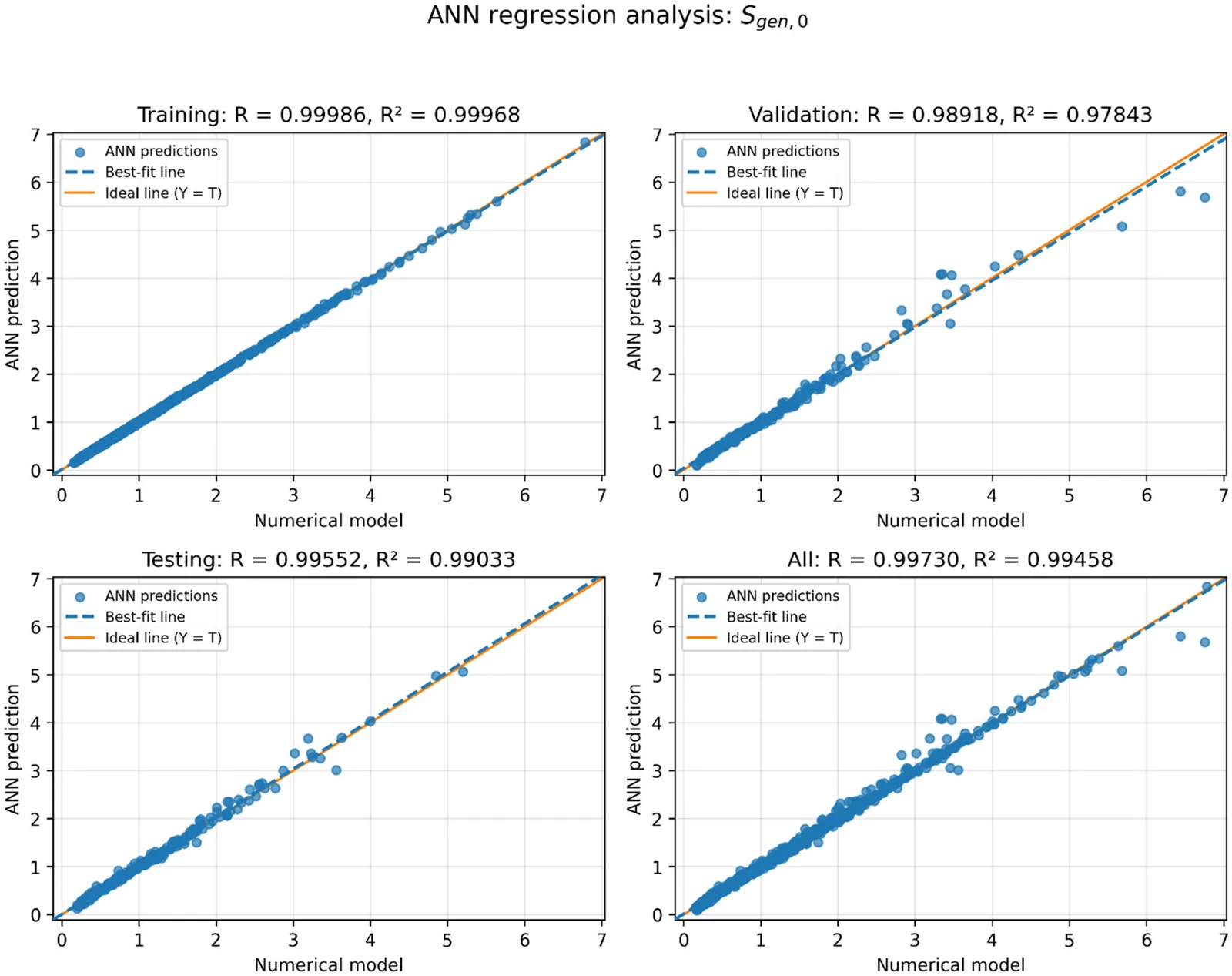
ANN regression analysis for entropy generation across the training, validation, testing, and overall datasets.

Overall, the ANN predictions agree closely with the numerical results for all three outputs, confirming the model's accuracy and reliable predictive capability.

Additional parametric results and ANN training-state diagnostics are provided in Supplementary Figures S1–S10.

## 8 Conclusion

In this work, the entropy generation by the convection of a Maxwell hybrid nanofluid (blood/Au–Cu) over an impermeable vertical cone embedded in a non-Darcy porous medium with the Cattaneo–Christov heat flux model is investigated. The numerical investigation is complemented by Sobol' global sensitivity analysis and ANN prediction to identify the influential parameters and assess the predictive capability of the developed model.

The most significant findings are summarized as follows:

- The magnetic field raised the temperature in the boundary layer while reducing fluid velocity due to the resistive Lorentz force.
- The porous-medium resistance strongly influences momentum and thermal transport by restricting fluid motion and modifying the associated heat-transfer behavior.
- Velocity and thermal slip alter the near-wall transport behavior and consequently affect the surface friction and heat-transfer characteristics.
- Entropy generation increases with the temperature-ratio parameter and Brinkman number, indicating greater contributions from thermal and viscous irreversibilities.
- The variation in the Bejan number reflects the relative contributions of heat-transfer and fluid-friction irreversibilities to the total entropy generation.
- The addition of Au and Cu nanoparticles improves the thermal transport characteristics of the base fluid; however, the associated variation in entropy generation should also be considered when selecting the nanoparticle concentration.
- Sobol' global sensitivity analysis identifies the porosity parameter K as the dominant factor governing the skin-friction coefficient and Nusselt number. For entropy generation, K and the temperature-ratio parameter make the major contributions within the investigated parameter ranges, while the remaining parameters exhibit comparatively smaller sensitivity contributions.
- The dominant influence of K provides a useful practical guideline for thermal-system design. Adjustment of the permeability and pore structure of the porous medium can be used to regulate the balance between flow resistance and heat-transfer performance. Hence, porous-medium characteristics should be given priority when selecting or optimizing operating conditions.
- The ANN surrogate models accurately predict the skin-friction coefficient and Nusselt number, with test R^2^ values of 0.99479 and 0.99642, respectively, demonstrating reliable predictive performance on unseen data.
- The ANN model for entropy generation achieves a test R^2^ value of 0.99033, demonstrating reliable prediction of thermodynamic irreversibility within the investigated parameter ranges.

Overall, the combined numerical, sensitivity, and ANN analyses identify the principal parameters governing momentum transport, heat transfer, and irreversibility. In particular, the sensitivity results provide a practical basis for selecting porous-medium characteristics to achieve an appropriate balance between heat-transfer enhancement and flow resistance.

### 8.1 Physical applications

The present study demonstrates the potential of combining Maxwell hybrid nanofluids with non-Fourier heat transfer in porous conical systems to regulate thermal transport and irreversibility characteristics. Using ANN surrogates provides a rapid and easy means of obtaining predictions of skin friction, Nusselt number and entropy generation without re-running the numerical computations. These capabilities are particularly applicable to nano-enhanced thermal management systems and compact heat exchangers involving viscoelastic fluids.

### 8.2 Prospects for the future and extensions of research

Although the ANN provides accurate predictions, its performance is limited by the numerical dataset used for training. Future research may consider physics-informed neural networks, larger parametric datasets, and unsteady, three-dimensional, and temperature-dependent models to extend the applicability of the predictive framework to more complex thermal systems.

## Data Availability

The raw data supporting the conclusions of this article will be made available by the authors, without undue reservation.
