# Supplementary material for: ANN-driven analysis of Cattaneo–Christov heat transfer and entropy generation in Maxwell hybrid nanofluid flow over a vertical cone
Source: Front Artif Intell. 2026 Sep 25;9:1936035. doi: 10.3389/frai.2026.1936035 (PMC13649697; doi:10.3389/frai.2026.1936035)
Supplement: Supplementary file 1 [file Data_Sheet_1.docx]

Fig. S1. Influence of the velocity slip parameter$\lambda$ on the temperature distribution

Figure S1 shows that the temperature profile decreases and the thermal boundary layer thins. This is because increased slip lowers the temperature inside the boundary layer by reducing the heat transferred from the hot cone to the fluid.

Fig. S2. Influence of nanoparticle volume fraction on velocity distribution

Figure S2 clearly shows that as the nanoparticle volume fraction increases, the velocity profile increases in the region close to the cone, with a higher peak velocity observed at larger values of ϕ. The addition of Au–Cu nanoparticles modifies the effective thermophysical properties of the hybrid nanofluid, which alters the momentum transport within the boundary layer. Farther from the cone surface, the curves intersect and the influence of ϕ changes as the velocity gradually approaches zero.

Fig. S3. Influence of nanoparticle volume fraction on temperature distribution

With an increase in the volume percentage of nanoparticles, Figure S3 shows that the temperature profile decreases with increasing nanoparticle volume fraction ϕ. The addition of Au–Cu nanoparticles modifies the effective thermophysical properties of the hybrid nanofluid, particularly its thermal conductivity, heat capacity, and viscosity. Within the considered parameter range, the enhancement in effective thermal conductivity facilitates energy transport through the boundary layer, resulting in a reduction in the dimensionless temperature and thermal boundary-layer thickness. Therefore, the observed temperature reduction arises from the combined modification of the effective properties rather than from thermal conductivity alone.

Fig. S4. Influence of nanoparticle volume fraction on entropy generation

Fig. S5. Influence of nanoparticle volume fraction on Bejan number

The effects of various volume fraction values on entropy generation and the Bejan number are shown in Figures S4 and S5. As the volume fraction increases, the Bejan number decreases, and the rate of entropy formation increases. Physically, the Bejan number decreases and entropy generation increases as nanoparticle volume fraction increases because the effects of heat transfer balance those of fluid and magnetic friction. For the considered range $\phi=0.02-0.06$, the decrease in the Bejan number indicates an increasing contribution of frictional irreversibility. The transition N_G_ > 0.5 moves toward the cone surface as $\phi$increases, indicating that frictional irreversibility becomes dominant earlier in the boundary layer.

Fig. S6. Influence of velocity slip parameter$\lambda$ and nanoparticle volume fraction on Nusselt number

As shown in Figure S6, the Nusselt number increases with the rise in the velocity slip parameter and nanoparticle volume fraction. The increase in nanoparticle volume fraction enhances the effective thermal conductivity of the hybrid nanofluid, while increasing velocity slip modifies the near-wall flow and strengthens the surface temperature gradient under the considered conditions, resulting in an increase in the Nusselt number.

Fig. S7. Influence of thermal slip parameter$\xi$ and nanoparticle volume fraction on skin friction

Figure S7 shows that skin friction decreases with the thermal slip parameter, resulting from reduced wall drag due to slip, but increases with the nanoparticle volume fraction because of the increase in effective viscosity.

Fig. S8. The training state on $R_{e_{x}}^{1/2}C_{f_{x}}$

Figure S8 presents the training state of the ANN model for predicting $R_{e_{x}}^{1/2}C_{f_{x}}$ . The gradient norm decreases substantially over the course of training, although intermediate fluctuations are observed as the network weights are updated. Its lower magnitude toward the final epochs indicates that the optimisation is approaching a stable solution. The learning rate is reduced stepwise from its initial value of $1\times10^{-3}$, allowing smaller parameter updates as training progresses. The validation-check history shows several resets when the validation performance improves, followed by a continuous increase after the best validation epoch. Training finally terminates when the early-stopping patience of 80 epochs is reached. Together, these trends show that the optimisation procedure progressively refines the network while early stopping prevents unnecessary continuation once the validation performance no longer improves.

Fig. S9. Training state of the ANN model for the Nusselt number, showing the gradient, damping parameter, and validation checks during training.

Figure S9 illustrates the training state of the ANN model for$R_{e_{x}}^{-1/2}Nu_{x}$**.** The gradient norm exhibits fluctuations during optimisation but shows an overall reduction toward the later epochs, indicating progressive adjustment of the network parameters. The learning rate decreases stepwise from $1\times10^{-3}$as the validation performance ceases to improve, enabling finer weight updates during the later stages of training. The validation checks repeatedly return to zero whenever an improvement occurs and subsequently increase continuously after the final improvement. Training terminates when the validation checks reach the prescribed early-stopping limit of 80. These features indicate that the learning-rate adjustment and early-stopping criterion effectively regulate the training process and limit further optimisation once no additional validation improvement is achieved.

Fig. S10. Training state of the ANN model for entropy generation, showing the gradient, damping parameter, and validation checks during training.

Figure S10 illustrates the training state of the ANN model for S_gen,0_. The gradient norm shows an overall decreasing trend with intermediate fluctuations, indicating progressive adjustment of the network parameters during optimisation. The learning rate is reduced in stages from its initial value of $1\times10^{-3}$ , allowing finer parameter updates as training progresses. The validation checks fluctuate during the intermediate epochs and eventually reach the prescribed patience limit of 80, at which point the training process is terminated. These characteristics indicate that the optimisation procedure progressively converges while early stopping prevents unnecessary continuation of the training process.

Fig. 22. Sobol’s sensitivity analysis showing the influence of model parameters on the skin-friction coefficient

Fig. 22 shows that the porosity parameter $K$ is clearly dominant, accounting for roughly 71% of the output variance at $Gr=0.5$and 67% at $Gr=1.5$. The next most significant parameter is the Deborah number $\Lambda$, which accounts for approximately 11% for $Gr=0.5$ and 15% for $Gr=1.5$. The first-order contributions of the remaining parameters are insignificant. The hierarchy of the total-order indices follows the same trend, with K remaining dominant, followed by the Deborah number and a relatively small contribution from the magnetic parameter M. The small $S_{T}$- $S_{1}$ gaps suggest that the response is governed mainly by direct effects, with modest interaction contributions.
